# Low dose of zearalenone elevated colon cancer cell growth through G protein-coupled estrogenic receptor

**DOI:** 10.1038/s41598-021-86788-w

**Published:** 2021-04-01

**Authors:** Emily Kwun Kwan Lo, Jetty Chung-Yung Lee, Paul C. Turner, Hani El-Nezami

**Affiliations:** 1 School of Biological Sciences, the University of Hong Kong, Hong Kong SAR, China; 2 School of Public Health, Maryland Institute for Applied Environmental Health, University of Maryland, College Park, MD USA; 3 Institute of Public Health and Clinical Nutrition, University of Eastern Finland, Kuopio, Finland

**Keywords:** Colorectal cancer, Cell growth

## Abstract

Colon cancer is one of the leading causes of cancer death worldwide. It is widely believed that environmental factors contribute to colon cancer development. Zearalenone (ZEA) is non-steroidal estrogenic mycotoxin that is widely found in the human diet and animal feeds. Most cancer studies of ZEA focused on estrogen sensitive cancers, while few focused on other types, such as colon cancer; despite the gastrointestinal tract being the first barrier exposed to food contaminants. This study investigated the stimulatory effects of ZEA on colon cancer cell lines and their underlying molecular mechanisms. ZEA promoted anchorage independent cell growth and cell cycle progression through promoting G1-to-S phase transition. Proliferative marker, cyclin D1 and Ki67 were found to be upregulated upon ZEA treatment. G protein-coupled estrogenic receptor 1 (GPER) protein expression was promoted upon ZEA treatment suggesting the involvement of GPER. The growth promoting effect mediated through GPER were suppressed by its antagonist G15. ZEA were found to promote the downstream parallel pathway, MAPK signaling pathway and Hippo pathway effector YAP1. Altogether, our observations suggest a novel mechanism by which ZEA could promote cancer growth and provide a new perspective on the carcinogenicity of ZEA.

## Introduction

Colorectal cancer (CRC) is the third most commonly diagnosed and second most deadly cancer in both males and females worldwide^1^. With improved diagnosis, medical care and knowledge of the risk factors, the survival rate of CRC patients has increased. Despite this, approximately 40% of patients survive for less than 5 years from diagnosis^2,3^; thus there is need to better understand other underlying reasons.

Zearalenone (ZEA) is frequently found in human and animal diet through consumption of fungal contaminated cereal products such as wheat, corn, oats and rye products^4^. ZEA is a non-steroidal estrogenic mycotoxin due to its structural similarities with 17-β-estradiol and therefore exert estrogenic activity via estrogen receptors ^5^. ZEA promoted breast and prostate cancer cell in vitro^5,6^; and ZEA metabolites were elevated in breast cancer patient in a study in Tunisia compared to controls^7^. However, most studies focus only on hormone sensitive cancers while neglecting the risk on non-hormone sensitive cancers, such as colon cancer. ZEA frequently contaminates grain based diets thus chronic gastrointestinal exposures, including in the colon, are predicted^8,9^. In a recent study, ZEA was found to significantly enhance the cell proliferation and cell migration in a colon cancer cell line HCT116^10^. Another recent study suggested that ZEA produce a proliferative state in porcine ex vivo intestine through the activation of WnT/β-catenin pathway and simultaneously suppressed TNF-b^11^. We have previously shown that ZEA could bind to GPER and ERa in breast cancer cell and promote the growth of cancer through MAPK/ERK pathway^12^. The ERK1/2 pathway have been known to contribute to the proliferation in colon, breast and prostate cancer^13,14^. As a downstream signalling branch of GPER, Hippo pathway has also been found to be frequently inactivated in several cancer^15^. MAPK/ERK and Hippo pathway are two independently parallel pathways which both act on the G1 phase restriction point in the cell cycle and promotes the progression from G1 to S phase leading to tumour proliferation. These two pathways work together and amplify one another^16,17^.

However, it is not yet clear if these pathways are involved in the effect of ZEA on colon cancer. Consequently, we hypothesized that ZEA could promote the CRC growth by the activation of ERK1/2 signalling and Hippo pathway through GPER. Specifically, we (1) investigated the effect of ZEA on proliferation of HT29, HCT116 and SW480 cells which have different mutation status of p53, (2) determined the role of GPER in the proliferative stage and (3) identified the downstream signal pathway involved during the proliferation.

## Results

### ZEA showed a dose-dependent effect on the proliferation of human CRCs

The in vitro cytotoxicity effect of ZEA was first evaluated on three different well characterized CRC cell lines namely, SW480, HT29, HCT116, and CCD841(normal cells) using MTT assay. Upon 72 h of treatment, the half inhibitory concentration (IC50) was 40.3 ± 1.1 μM (SW480), 48.4 ± 1.1 μM (HT29), 29.8 ± 1.1 μM (HCT116) and 62.6 ± 1.1 μM (CCD841). ZEA did not show any significant effect on any of the CRCs cell viability at the three lowest doses, < 12 μM (Fig. S1), Subsequently, CRC cell proliferation assays dosed ZEA at sub-cytotoxic concentrations. It is known that both SW480 and HT29 harbor p53 mutation^18^. These cell lines were more resistant to the cytotoxicity effect of ZEA in this study when compared to the p53 wild type HCT116. Moreover, mutation of p53 (a tumour suppressor) was found in nearly 60% of colon cancer patients^19^, therefore, these two cell lines were selected as the cell model for all the following experiments.

**Figure 1:**
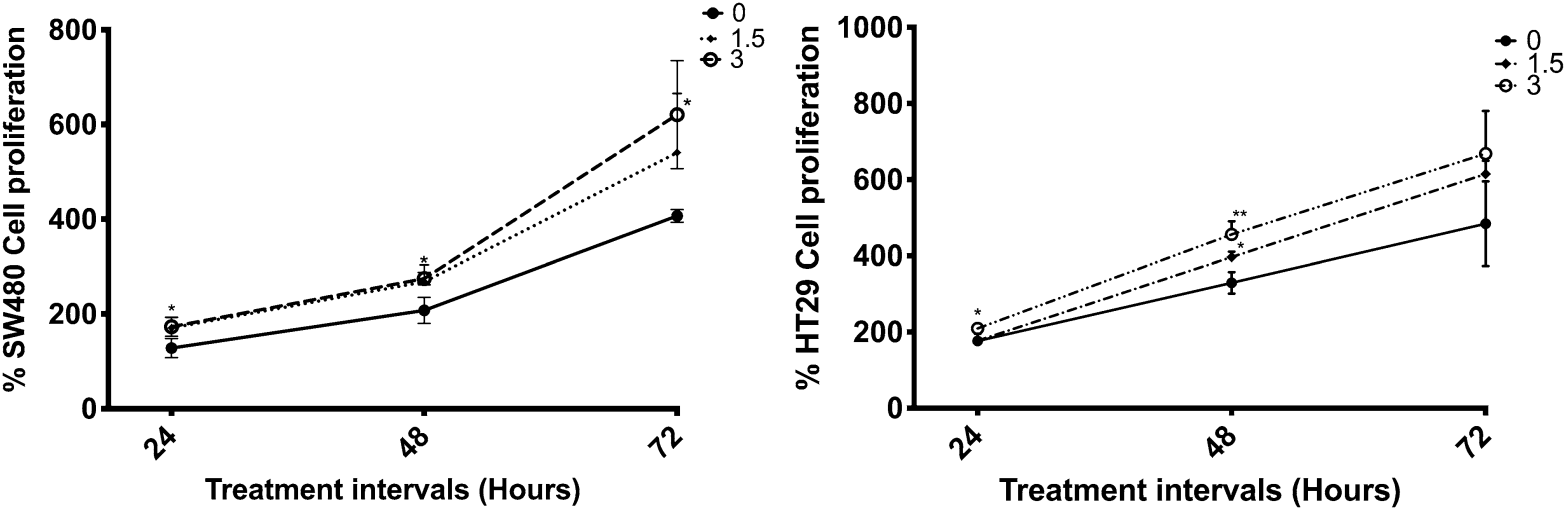
ZEA promotes the growth of human CRC cells. Cultured CRC cells (SW480, HT29) were treated with 0–3 μM ZEA for 24, 48 and 72 h. CRCs were then trypsinized and fixed with trypan blue solution. Cell counting was carried out using LUNA Automated Cell Counter. Results shown are mean ± SEM, n = 3. *p < 0.05 and **p < 0.01 compared to control.

CRC cell growth was measured in untreated and cells treated with 1.5 and 3.0 μM ZEA. Treatment of 3 μM ZEA significantly increased cell proliferation in SW480 at 24, 48 and 72 h of treatment (45%, 67%, and 214%, (p < 0.05), respectively compared to untreated controls); in HT29 at 24 and 48 h of treatment (33% (p < 0.01) and 127% (p < 0.05), respectively) (Fig. 1)., and in HCT116 at 24 h of treatment (46% (p < 0.05)) (Fig. S1B). In flow cytometry experiments, ZEA significantly increased the percentage of BrdU positive cell i.e. S phase cells (p < 0.001) and decreased in G0/G1 phase cells (p < 0.05) in SW480 (Fig. S2).

### Zearalenone caused GPER protein retention in CRC cells

**Figure 2:**
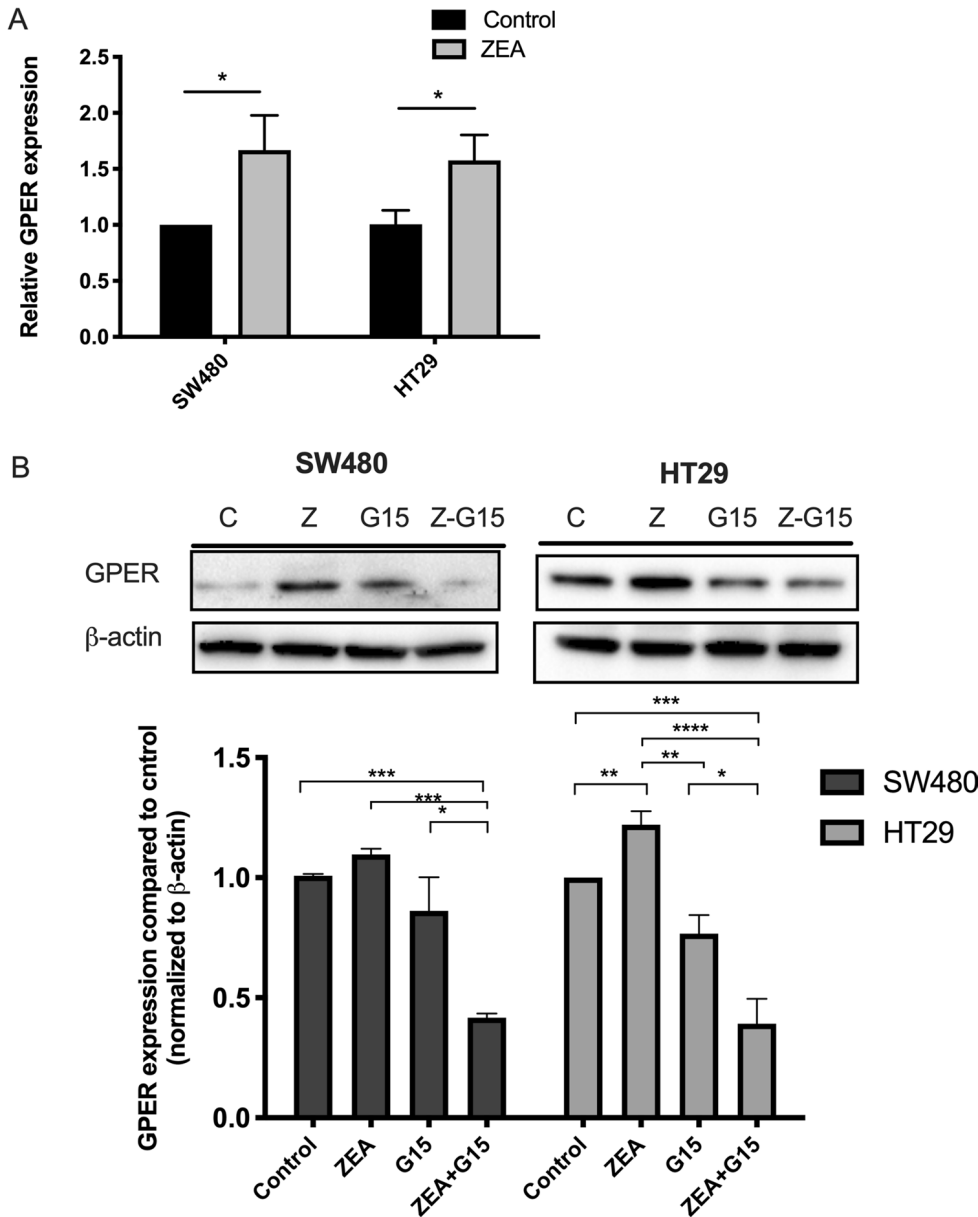
ZEA increases GPER expression in CRC cell. (**A**) RT-qPCR analysis of GPER gene expression in SW480 and HT29 cells with and without 3 μM ZEA was investigated. (**B**) Western blot analysis of GPER in SW480 and HT29 with and without G15 treatment was investigated. Results shown are mean ± SEM, with n = 3. *p < 0.05, **p < 0.01, ***P < 0.001 and ****p < 0.0001.

The basal expressions of GPER, ERα in the SW480 and HT29 were first determined. ERα is absent in both colon cancer cell line (Figure S3B). SW480 had a significant higher GPER (p < 0.0001) protein expression when compared to HT29 (Fig. S3A). Upon 24 h of ZEA treatment, the mRNA expression level of GPER increased in both SW480 (p < 0.05) and HT29 cells (p < 0.05) when compared to control (Fig. 2A). The GPER protein expression was significantly increased in HT29 cells (p < 0.05) (Fig. S3C) while GPER mRNA was significantly increased in SW480 cells when compared to control.

To determine whether there was a positive feedback loop involved in the increased expressions in GPER, SW480 and HT29 cells were treated with 3 μM ZEA, 5 μM G15 (GPER antagonist) and 3 μM ZEA + 5 μM G15. The concentration of G15 is based on previous study by Bustos et al. who showed GPER to be a mediator for the pro-tumorigenic effect of E2^20^. After 24 h, there was no difference in GPER expressions between control and G15 groups. ZEA group significantly increased the expression of GPER compared to the control group in HT29 cells (p < 0.05). The addition of G15 significantly eradicated the stimulatory effect of ZEA in both SW480 (p < 0.001) and HT29 (p < 0.0001) cells (Fig. 2B).

### ZEA, acting through GPER, promoted CRC growth via cell cycle shift and anchorage independent growth

**Figure 3:**
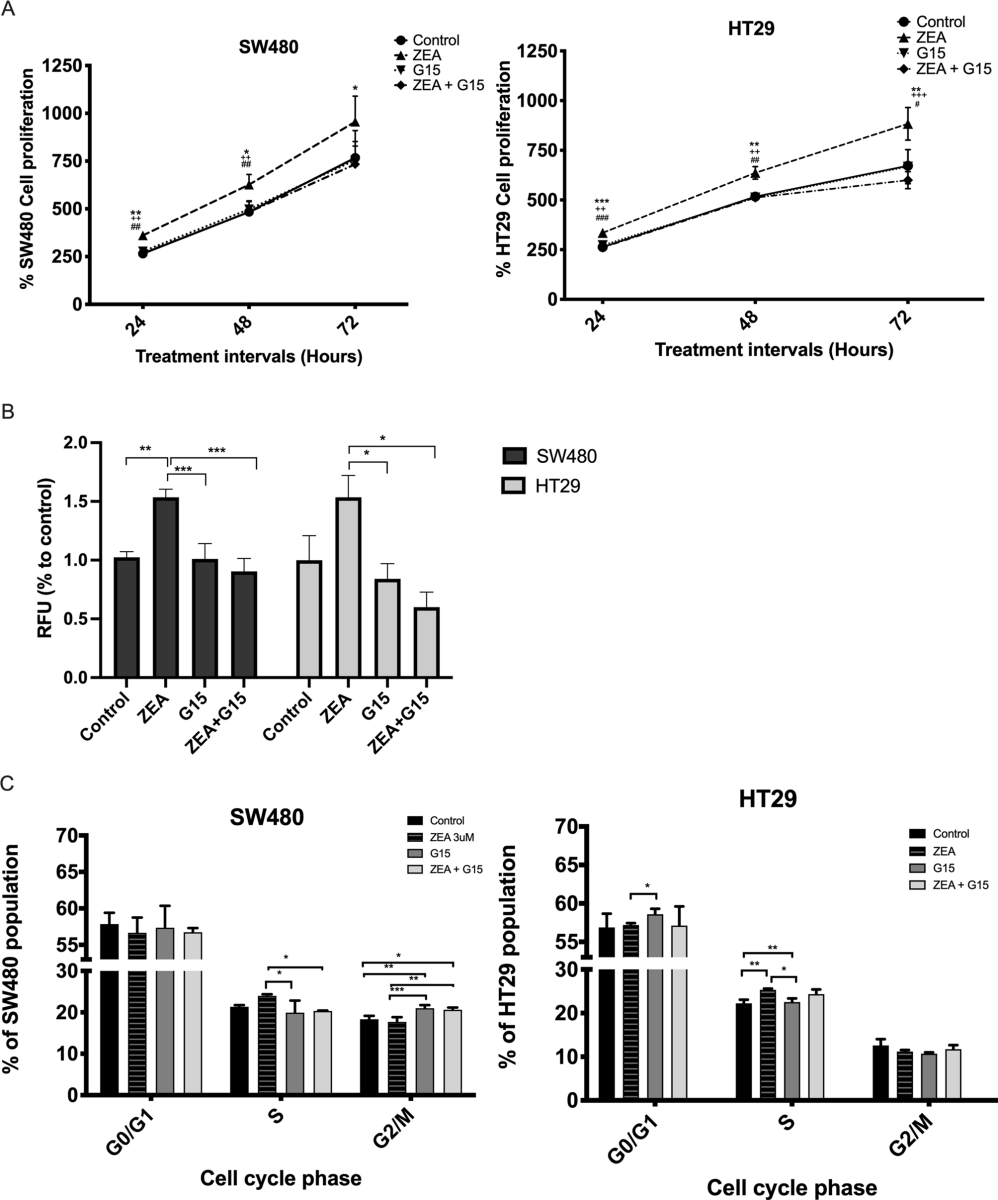
Zearalenone promoted the growth of human CRC cells. (**A**) SW480 and HT29 cells were incubated with 3 μM ZEA with or without G15 (GPER antagonist) for 24, 48 and 72 h. CRCs were then trypsinized and fixed with trypan blue solution. Cell counting was carried out using LUNA Automated Cell Counter. *p < 0.05, **p < 0.01 and ***p < 0.001 ZEA compared to control. ^+^p < 0.05, ^++^p < 0.01 and ^+++^p < 0.001 G15 compared to ZEA. ^#^p < 0.05, ^##^p < 0.01 and ^###^p < 0.001 ZEA + G15 compared to ZEA. (**B**) Soft agar transformation assay was performed. RFU refers to Relative fluorescent units. (**C**) Cell cycle analysis of SW480 and HT29 co-incubated 3 μM ZEA with or without G15 for 24 h. The percentage of S phase, G0/G1 and G2/M are shown in the figures. Results shown are mean ± SEM, with n = 3. *p < 0.05, **p < 0.01 and ***p < 0.001.

To determine if GPER had functional involvement in ZEA proliferative effect on CRC cells, live cell counting was performed. After 24 and 48 h, ZEA significantly promoted cell growth in both SW480 (p < 0.05) and HT29 (p < 0.01) cells when compared to control. The addition of G15 significantly attenuated the proliferative effect from ZEA (p < 0.01) when compared to ZEA alone (Fig. 3A).

One of the important hallmarks of cancer is to be able to have anchorage independent growth i.e. the ability to proliferate unattached to extracellular matrix and neighboring cells. To investigate the anchorage independent growth, the soft agar colony formation assay was performed. The results from soft agar colony formation assay were consistent with cell number counting. Following seven days of treatment, ZEA increased the colony forming ability of CRC by 51% (SW480, p < 0.005) and 54% (HT29) when compared to control. The addition of G15 significantly diminished the cell proliferation effect from ZEA (Fig. 3B).

To confirm the GPER involvement on ZEA induced S phase cell cycle progression, the proliferation of SW480 and HT29 cells were determined by BrdU staining. The number of BrdU positive cells (S phase cells) was significantly increased upon ZEA treatment in HT29 cells (p < 0.001) compared to control while there was no significant difference between ZEA treated and co-treatment (ZEA + G15). In SW480, the addition of G15 significantly lowered numbers of S phase cells (p < 0.05) compared to ZEA treatment alone. ZEA + G15 mix promoted the cell cycle arrest in G2/M phase in SW480 cells compared to control or ZEA treatment alone (Fig. 3C). This could be explained by the higher expression of GPER in SW480, and therefore the antagonistic effect was more profound (Fig. S3A).

### ZEA promoted proliferation marker expression through GPER

**Figure 4:**
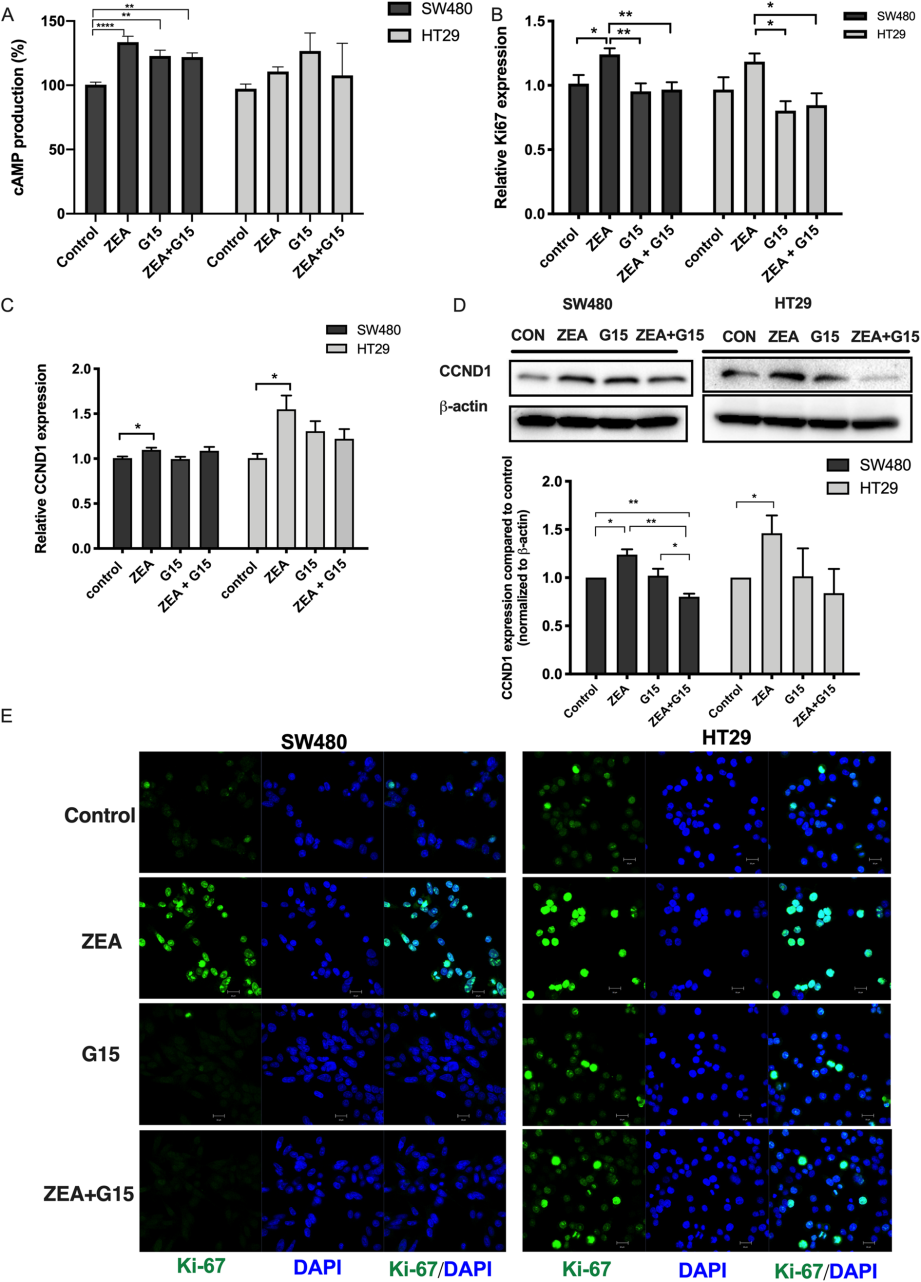
ZEA increased the expression of proliferation marker. RT-qPCR analysis of (**A**) CCND1 and (**B**) Ki67 mRNA expression of SW480 and HT29 incubated with 3 μM ZEA with or without G15 (GPER antagonist). (**C**) cAMP production induced by 3 μM ZEA in the absence or presence of 5 μM G15. cAMP production was analysed by comparing the cAMP production to control group as 100%. (**D**) Western blot analysis of CCND1 protein expression, (**E**) Ki-67 expression was determined by immunofluorescence staining for Ki67 (green) along with DAPI for DNA (blue). Scale bar, 20 μm. Results shown are mean ± SEM, with n = 3. *p < 0.05 and **p < 0.01.

To elucidate whether ZEA could promote the GPER mediated rapid signaling, cAMP was measured in SW480 and HT29 cells. ZEA showed a significant increase in cAMP production for SW480 compared to control (p < 0.001). G15 was also observed to increase cAMP levels. The predicted increase in cAMP if purely additive would equate to an overall % increase of 56% and 37% respectively, however in both cell models there was an overall reduction in cAMP with ZEA + G15, suggestive that G15 may be reducing ZEAs promotion of cAMP (Fig. 4A).

To further confirm the role of GPER in proliferative effect of ZEA, the cell proliferative marker, cyclin D1, a G1/S phase transition regulator, was measured. ZEA significantly increased the RNA and protein expression in both SW480 (p < 0.05) and HT29 (p < 0.05) cells (Fig. 4C-D). The effect of ZEA on CCND1 protein expression was significantly reduced when co-incubated with G15 in the SW480 cells (p < 0.01).

Next, the effect of ZEA on the expression of the nuclear proliferation marker, Ki67 was examined. The Ki67 mRNA expression was significantly increased upon treatment in SW480 cells (p < 0.05). The up-regulation in Ki67 was found to be reduced by G15 in both SW480 (p < 0.01) and HT29 (p < 0.05) cells (Fig. 4B). Immunostaining further showed that there was an increase in Ki67 positive cell (green fluorescence) upon 24 h of ZEA treatment in both SW480 and HT29 cells. G15 reduced the promotive effect on Ki67 fluorescence signal under ZEA treatment in both SW480 and HT29 cells (Fig. 4E). The blockage of G15 on ZEA effect was more profound in SW480 than HT29, which correlated with its higher basal expression in GPER (Fig. S3A). The high expression of CCND1 and Ki67 indicated a shift in the cell cycle.

### ZEA activated the ERK1/2 pathway

**Figure 5:**
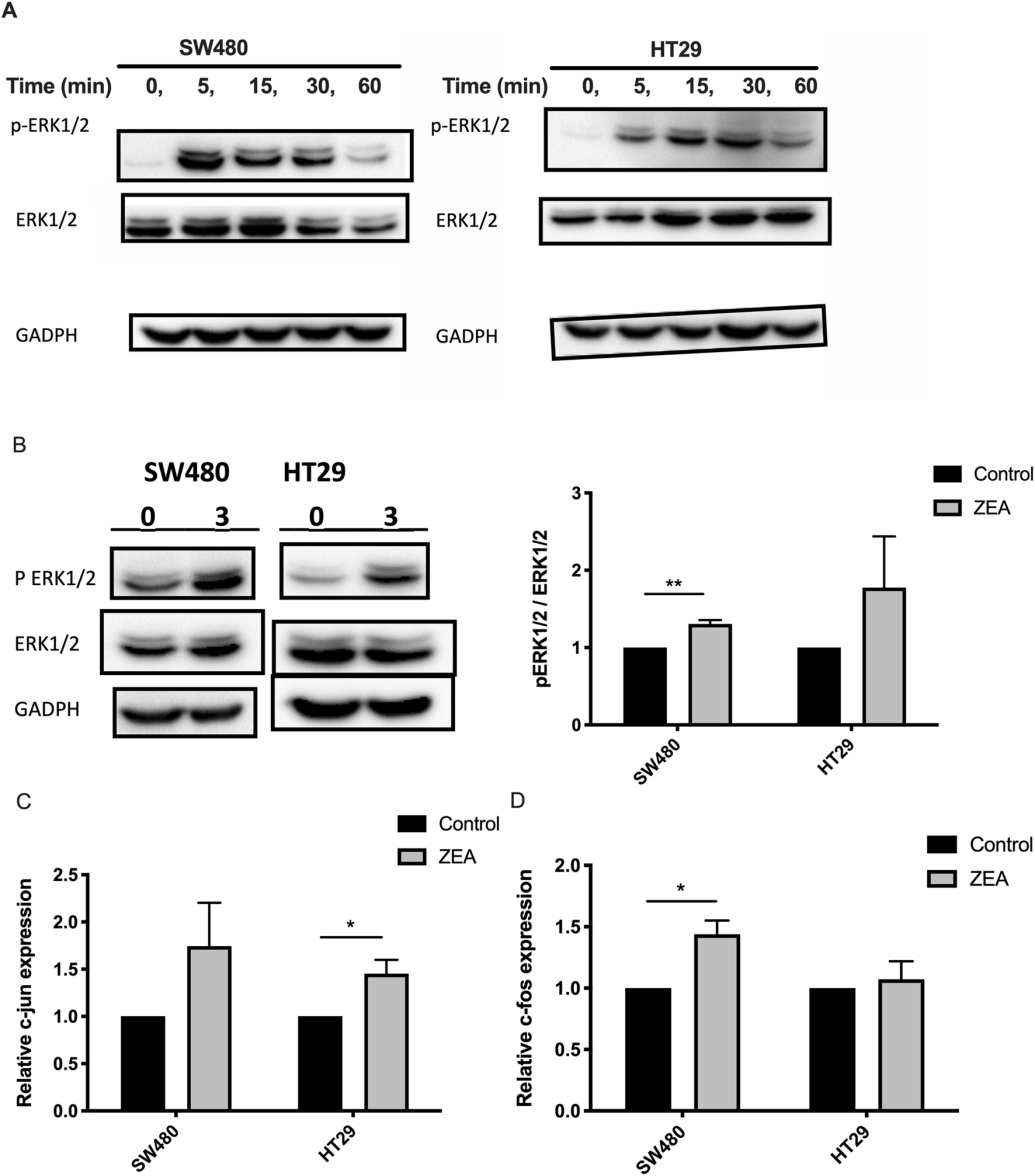
ZEA triggered the MAPK-ERK pathway. (**A**) Western blot analysis of the total and phosphorylation of ERK1/2 protein expression after CRC cells treated with 3 μM ZEA (A) in 1 h, and (**B**) at 15 min. (B) RT-qPCR analysis of downstream marker c-jun (**C**) and c-fos (**D**) expression in SW480 and HT29 incubated with and without 3 μM ZEA. Results shown are mean ± SEM, n = 3. *p < 0.05 and **p < 0.01.

The mechanism by which ZEA induced cell proliferation was further investigated by examining ERK1/2 signaling. Western blot expressions showed that ZEA induced the phosphorylation of ERK1/2 in a time-dependent manner (Fig. 5A). In SW480 and HT29, p-ERK1/2 was evident at 5 min and peaked at 15 min and significantly diminished by 60 min, therefore 15 min was subsequently used as the treatment period for additional ERK1/2 pathway analysis. ZEA significantly increased the ratio of pERK1/2 / tERK1/2 in SW480 (p < 0.01) (Fig. 5B). The activation was further demonstrated through the increased mRNA expression on downstream marker c-jun and c-fos in SW480 and HT29 cells. After 24 h of ZEA treatment, SW480 cells showed significantly increased c-fos expression (p < 0.05) while HT29 cells had significantly increased expression of c-jun (p < 0.05) compared to control (Fig. 5C-D).

### ZEA induced YAP1 nuclear localization via the GPER pathway

**Figure 6:**
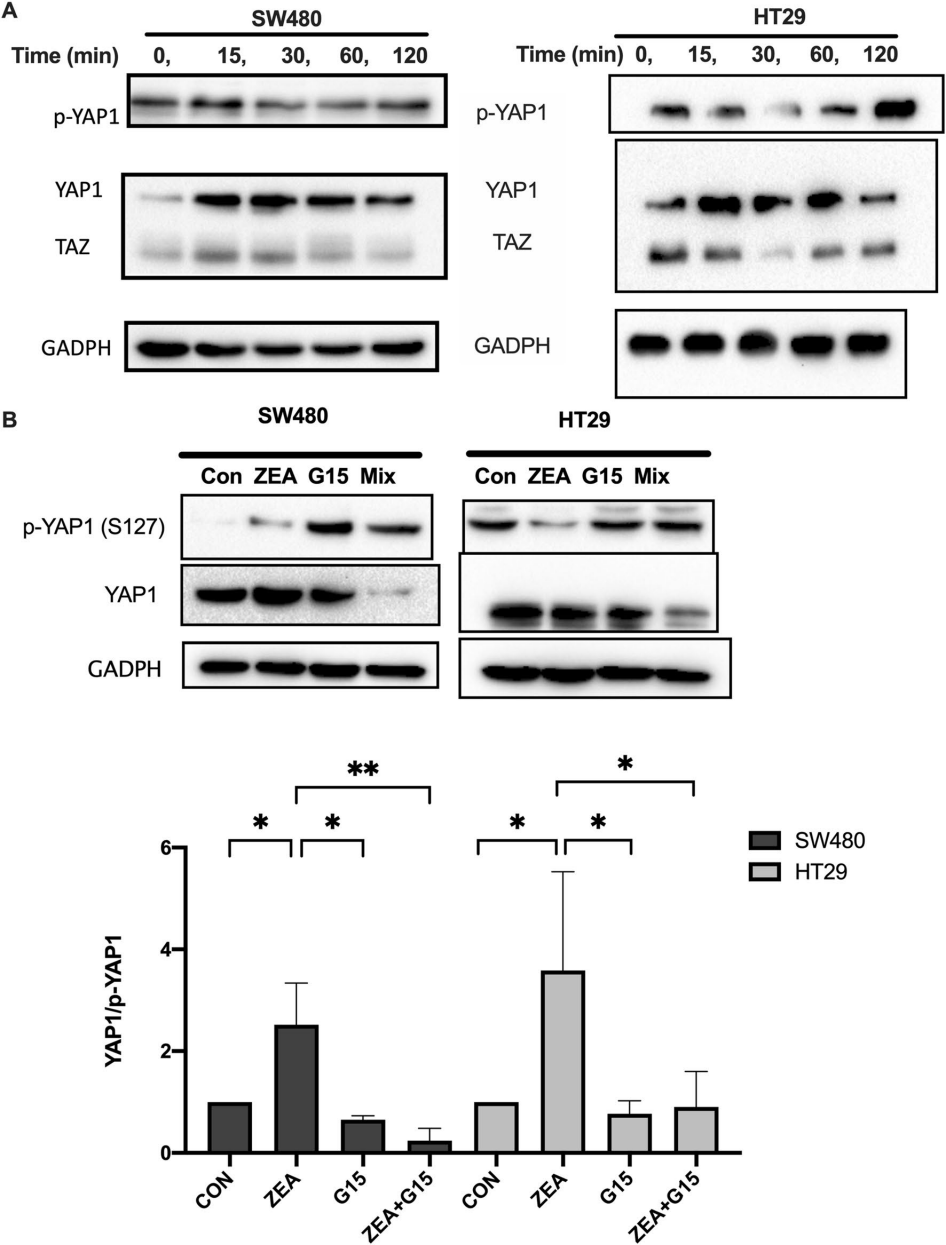

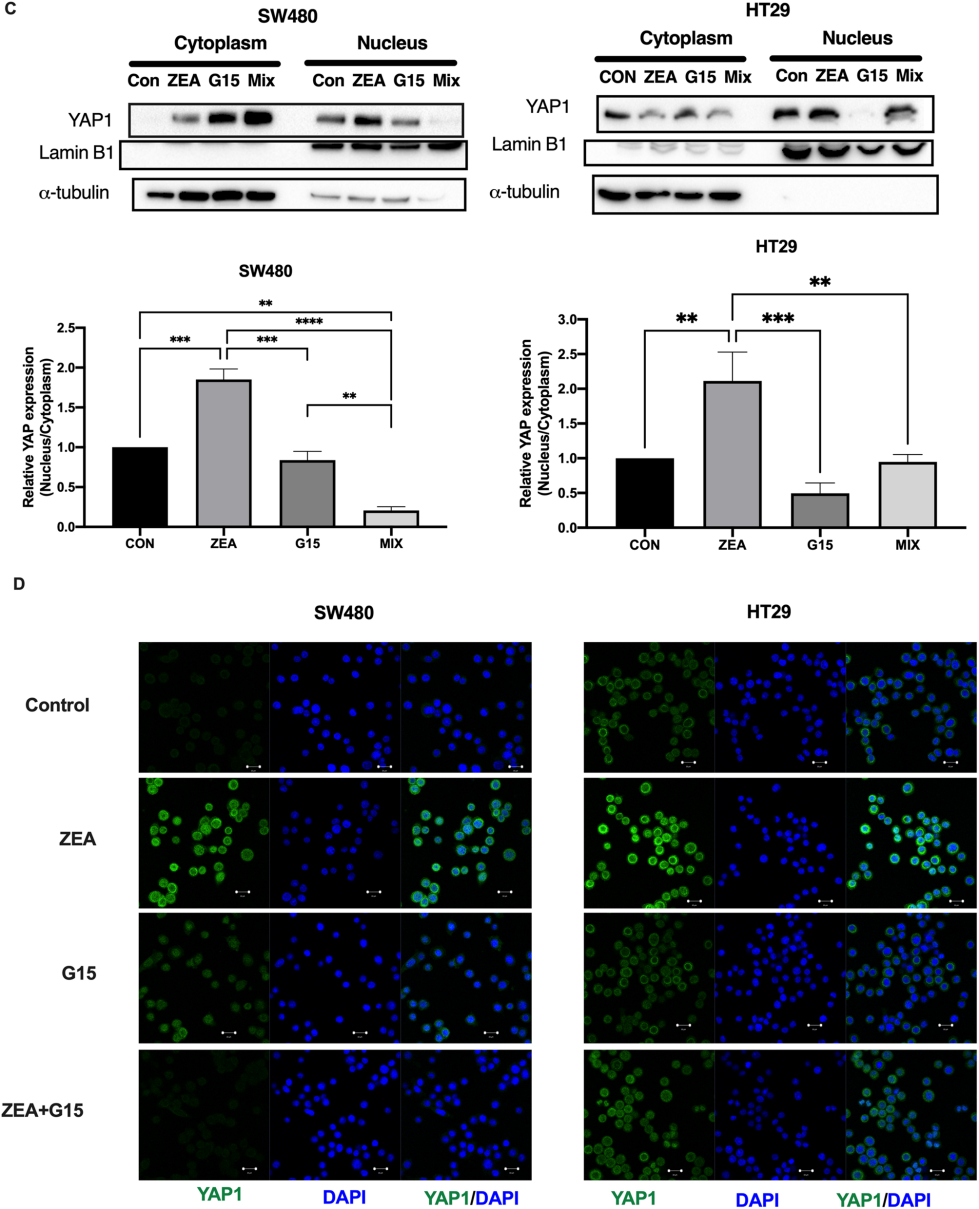
ZEA promoted YAP nuclear localization and activity through GPER in human CRC cells. (**A**)Western blot analysis of the total and phosphorylation of YAP1 and TAZ protein expression after CRC cells treated with ZEA. Western blot analysis of YAP expression in total (**B**), cytoplasmic and nuclear fraction (**C**) of SW480 and HT29 cells after incubated with 3 μM ZEA with or without G15 (GPER antagonist).GADPH a-tubulin and lamin B1 were used as a loading control for total, cytoplasmic and nucleus protein. (**D**) YAP nuclear localisation was determined by immunofluorescence staining for endogenous YAP1 (green). Nuclei was visualized using DAPI for DNA (blue). Results shown are mean ± SEM, n = 3. *p < 0.05, **p < 0.01 and ****p < 0.0001.

At time zero YAP1 was fully phosphorylated (p-YAP1). There was a trend of a decreased ratio of p-YAP1 to the total YAP1 (p-YAP1/t-YAP1) in both SW480 and HT29 cells; the dephosphorylation was found to be more profound after 30 min and 60 min of treatment (Fig. 6A). Since there was no significant difference between the two time points, 30 min was selected to further examine the Hippo pathway. Upon 24 h of ZEA treatment, YAP1 mRNA expression was significantly up-regulated in SW480 cells (p < 0.05), while TAZ mRNA expression was found to be up-regulated in both SW480 (p < 0.01) and HT29 (p < 0.05) cells compared to control (Fig. 6B).

To investigate whether ZEA promoted the nuclear translocation of YAP, the expression was analysed by immunofluorescent staining. We observed the nuclear accumulation of YAP in both SW480 and HT29 cells by ZEA and the effect was blocked by the co-treatment of G15 (Fig. 6D). This was also confirmed by the significantly higher nuclear to cytoplasmic ratio of YAP expression in ZEA treated SW480 (p < 0.001) and HT29 cells (p < 0.01). The co-treatment of antagonist G15, significantly reduced nuclear to cytoplasmic ratio of YAP expression (p < 0.01) when compared to control in SW480 (p < 0.0001) and HT29 (p < 0.01). Collectively, we found that ZEA induced the activation of YAP1 nuclear localization.

To determine whether the cytoplasmic YAP was phosphorylated, we extracted the nucleus and cytoplasmic extracts of the treated cells. In SW480 cells, ZEA significantly increased the nucleus protein expression in YAP1 (p < 0.001) when compared to control. This indicated that the nuclear localization of YAP1 is correlated with GPER activation. Similarly in HT29 cells, co-treatment with G15 was found to increase the pYAP/tYAP ratio, which suggest GPER involvement in the dephosphorylation of YAP1 (Fig. 6C). The increased unphosphorylated YAP1 protein expression suggest the activation of Hippo-YAP nuclear localisation.

## Discussion

This study is the first to investigate the effect of ZEA on cell growth and cell cycle progression on a panel of CRC cells, and its specific involvement of GPER and the downstream pathway. To date, most of ZEA’s carcinogenic potential studies have been focused on its effect on hormonal dependent cancer. However, there is lack of data on the effect of ZEA on non-hormone sensitive cancer cell types such as those from colon. Given the frequent dietary consumption of grains contaminated with ZEA, frequent and chronic exposure through diet is likely to expose colon cells to ZEA^21,22^. Our data demonstrate that ZEA promotes the proliferation and anchorage-independent growth in CRC cells. The ability for anchorage- independent growth is a pre-requisite for colon cancer to be metastatic. ZEA also promoted DNA synthesis and increased percentage of cells in proliferative cell cycle phase, S phase and G2-M phase. The induced cell cycle progression was supported by upregulating cell proliferative markers cyclin D1 and Ki67. This is in agreement with previous study on ZEA cell proliferation effect on colon, breast and prostate cancer^6,10,12^. The loss of cell cycle control is one of the characteristics of cancer development. Cyclin D1 is a key regulator for G1 to S phase progression while Ki67 is expressed in proliferative cells from G1 to M phase progression. The over-expression of cyclin D1 shortens the G1 phase and subsequently accelerate the cellular proliferation. Several studies indicated an overexpression of cyclin D1 is associated with tumorigenesis in CRC^23,24^. The high expression of cyclin D1 and Ki67 is related to a significant shorter overall survival in colon cancer patients^25–27^.

In this study, we confirmed the involvement of GPER in ZEA induced proliferation. There are three types of estrogenic receptors, ERα, ERβ and GPER. ERβ is the predominate ER in colon tissue, while ERα is absent^28–30^. ERβ is known to have tumor suppressor effect, while loss of ERα and β are observed in the malignant process in CRC. The role of GPER in colorectal cancer has been a controversial topic. It is suggested GPER to be a tumour promotor and to mediate CRC proliferation upon estrogen exposure^31^. Another study suggested that the activation of GPER inhibited CRC cells proliferation^32^.

We hypothesized that colon cancer proliferation by ZEA might be due to the loss of ERβ accompanied by the activation of GPER^20,33^. Consequently, the RNA and protein expression of GPER and ERβ were evaluated. Our data indicated high ERβ mRNA expression and undetectable ERα expression in CRC cells. Since GPER is found to promote CRC growth through mediating estrogenic activity in CRC cells^31^, the higher basal GPER expression might be one of the reasons for the higher SW480 cells growth. We suspected that the low basal level of GPER could explain the inability of G15 to reduce the cAMP level in HT29. Upon 24 h of ZEA treatment, the protein expression of GPER was elevated in both SW480 and HT29. It is therefore postulated that the upregulation of GPER could potentially be involved in sustaining the estrogenic proliferative signal. GPER agonist 17-β-estradiol have been shown to bind to GPER. Indeed, proliferation of human CRC have been found to be associated with the enhanced estrogen metabolism and GPER protein expression^31^. The involvement of GPER in ZEA’s proliferative effect was further confirmed by co-incubating CRCs with GPER antagonist (G15) on cell growth. The inhibition on GPER significantly diminished the induced proliferation and anchorage-independent growth. Similar finding was reported for GPER agonist Nonylphenol which was found to increase GPER protein expression but abolished upon co-treatment of G15 in CRC cell line SW480 and COLO205^34^. The inhibition also significantly decreased the expression of cell proliferation marker CCND1 and Ki67.

MAPK play an active role in cancer progression by regulating cell differentiation, proliferation and migration. Studies have demonstrated that the activation of MAPK attributes to the therapeutic resistance for cancer^35^. It is evident that extracellular-signal-Regulated Kinase ½ (ERK1/2) is one of the key downstream pathway of GPER and contributes to cell proliferation, survival and cancer progression^36^. On the other hand, it has been shown that dysregulated Hippo pathway is associated with cancer development such as colon, lung, liver, prostate and ovarian cancer development^37^. YAP was found to be overexpressed in 53% of CRC occurrences and are mostly translocated to the nucleus^38^. The Hippo pathway plays a crucial role in cancer growth. The dephosphorylation of YAP/TAZ trigger their translocation from the cytoplasm to the nucleus, promote the oncogenic downstream signalling pathway and hence activate the target genes expressions. Previous studies have shown that the nuclear localization could lead to cell proliferation^39,40^. Moreover, activation of YAP/TAZ showed to increase proliferation through downstream effector including Wnt and MAPK signalling. Additionally, it has been reported that estrogen could activate YAP-TAZ downstream effector, CYR61 through GPER^41^.

In this study, ZEA was found to activate ERK1/2 pathway which is consistent with the previous findings in our lab on breast cancer cells^12^. Recent report proposed that MAPK pathway regulate YAP activity through inhibition of phosphorylation on Lats. This would lead to reduced activity on its phosphorylation in YAP and subsequently, promote the nuclear translocation^42^. In non-small cell lung cancer, the inhibition on ERK1/2 was found to down-regulate YAP1^43^. In our study, ZEA was found to promote nuclear localization of YAP1. We observed increased YAP1 and TAZ mRNA, and protein suggesting the progression of cancer. Zhou et al. also reported augmented YAP1 and TAZ mRNA levels in colon cancer tumor samples when compared with healthy colon tissues^41^. Our results are also consistent with a study on mouse ovarian granulosa cells which showed ZEA to activate YAP1/TAZ signalling^44^. Of note, in this study, TAZ expression was very low and did not response to ZEA treatment in HT29 cells. The trend was opposite to what we have found in SW480 cells, which could be explained by the much lower abundancy in GPER expression. It is possible that the later increase in mRNA expression in YAP and TAZ in HT29 upon 24 h ZEA treatment is correlated with the increased expression of GPER, which is a regulator of Hippo pathway^45^. To have a deeper understanding on the involvement of Hippo pathway in ZEA’s effect, we studied the phosphorylation status of YAP1 in total cell and the YAP level in cytoplasmic and nucleus protein extracts. Increased YAP1 protein expression in the nuclear protein extract by ZEA was observed. The involvement of GPER in ZEA effect was further confirmed through the abolishment of nuclear localization by the G15. The YAP expression was lowered when co-treated with G15. As a consequence, it potentially led to protein degradation of YAP1 and become inhibited to be translocate into the nucleus^46^.

In this study, we found that CRC cell lines harboring p53 mutation (HT29 and SW480 cells) are more susceptible to the proliferative effect of ZEA. p53 is a tumour suppressor and plays a crucial role in directing DNA damaged cells to apoptosis. Mutation of p53 is known to be associated with colon cancer onset and progression. The mutation is found in nearly 60% of the colon cancer patients^19^. The response from HT29 and SW480 cells was slightly different due to their difference in both morphology and expression level in differentiation marker^47^. Furthermore, p53 is also known to be linked to YAP1 activity. Mutated p53 could bind to YAP1 and form a pro-tumorigenic crosstalk, and subsequently promote the growth of tumor cells and migration^48^.

## Conclusions

Collectively, our results suggest that ZEA stimulated DNA synthesis, cell cycle shift, colon cancer proliferation and anchorage-independent growth through its activation to GPER in vitro. The growth promotion was mediated by the activation on ERK1/2 and YAP1/TAZ signaling. Our findings suggest that ZEA could stimulate active colon cancer cells, and thus are potentially a threat to colon cancer patients, these cell culture derived data merit further investigation in animal feeding studies.

## Materials and methods

### Cell culture and treatment

Two human CRC cell lines, HT29 and SW480 cells were obtained from American Type Culture Collection (ATCC, Manassas, VA, USA). The three cell lines were selected based on their mutation status of p53. The mutation status of the cell lines is detailed in Table S1. The normal human colon cell line CCD-841 was chosen as a control to compare the proliferation by ZEA. These cell lines were maintained in complete medium containing Dulbecco’s Modified Eagle’s Medium (DMEM) supplemented with 10% fetal bovine serum (FBS) (Gibco, NY, USA), and incubated at 37 °C in an atmosphere of 95% air and 5% CO^2^ humidified incubator. The medium was changed every two days, and the cells were passaged using TrypLE Express (Gibco, NY, USA). All the experiment was conducted within 10 passages. To eliminate the potential interference from the FBS which contains estrogen, and phenol red which binds to ERs, we replaced the regular medium with phenol red free DMEM with charcoal stripped FBS (Gibco). For each experiment, the cells were steroid starved with phenol red free DMEM medium supplemented with 5% charcoal stripped FBS for 48 h before treatment. The cells were treated with ZEA (Sigma-Aldrich, MO, USA) and G15 (GPER antagonist) (Cayman Chemical, MI, USA) with concentrations described below. Both ZEA and G15 were dissolved in 100% DMSO and stored at -20˚C . The concentration of DMSO in the treatment was maintained at 0.5%.

### Cell viability assay

Cell viability was measured by the colorimetric 3-(4, 5-dimethylthiazolyl-2)-2, 5-diphenyltetrazolium bromide (MTT) assay. The cells were plated in 96-well plate with 5000 cells per well in steroid starving medium with ZEA concentration ranging from 0–100 μM for 24, 48, 72 h. Afterwards, MTT solution (0.5 mg/ml) (Sigma, MO, USA) was added to each well and incubated for additional 2 h and then, DMSO was added after removing the supernatant. The absorbance was measured at 570 nm by a Multiskan Go microplate spectrophotometer (Thermo Fisher Scientific, MA, USA).

### Proliferation assay

The cells were plated in a 12-well plate at 1 × 10^5^ cells per well in steroid starving medium. After 24, 48, 72, and 96 h of ZEA treatment, the cells in each treatment were harvested, stained with 0.4% trypan blue (Gibco, NY, USA) and counted with LUNA automated cell counter (Logos Biosystems, Gyeonggi-do, Korea).

### Colony formation assay

The colony formation of the cells was conducted using CytoSelect 96-well cell transformation assay kit (Cell Biolabs, San Diego, CA) according to the manufacturer’s instructions. In brief, cell suspension (7.5 × 10^3^) was prepared in 0.4% agar with DMEM and seeded in 96 well plates pre-coated with 0.6% agar solution. The treatment was added to each well after solidification of the cell agar. The cells were lysed after seven days of incubation. The DNA were stained with CyQuant GR dye and determined by Victor X4 fluorescent plate reader (PerkinElmer, CT, USA) using a 485/520 nm filter.

### Flow cytometry (FACS) analysis

The cells were plated in 60 mm dish with 1 × 10^6^ cells per dish. The analysis was performed using FITC BrdU flow kit (BD Pharmingen, San Diego, CA, USA) following manufacturer’s instructions. In brief, the cells were incubated with 10 μM bromodeoxyuridine (BrdU) for 45 min prior to harvesting. After trypsinization, they were washed in ice cold staining solution. The cells were fixed and permeabilized with Cytofix buffer and Cytoperm buffer, followed by washing with Perm/wash buffer. The cells were then treated with DNase for one hour and stained with FITC-conjugated anti-BrdU antibody and 7-ADD. After staining, the cells were counted using BD FACS AriaIII (Becton Dickinson, CA, USA). FACS data were then analyzed using the FlowJo software (Tree Star, Inc., California, USA).

### cAMP measurements

The cAMP production was determined using Hunter & Glass method with modification ^49^. In brief, 2 × 10^4^ HT-29 and SW480 cells were seeded in 96-well culture plates and incubated for 24 h. The cells were serum starved with DMEM containing 5 mg/kg BSA and 0.5 mM 3-isobutyl-1-methylxanthine (IBMX) for 30 min. Cells were then treated for 20 min. Reaction was terminated by removing the treatment mix and by adding ice cold stimulation buffer. Intracellular cAMP was subsequently determined using LANCE cAMP Detection Kit (Perkin Elmer, CT, USA) following the manufacturer’s instruction***.***

### RNA extraction and Real time reverse transcription polymerase chain reaction (RT-qPCR)

Total RNA was extracted with RNAiso plus (Takara, Kyoto, Japan). It was then centrifuged with chloroform, precipitated with isopropanol, and washed with 70% ethanol. RNA pellets were then dissolved in DEPC water. The purity of RNA concentration was determined with a NanoDrop 2000 spectrophotometer (Thermo Scientific, DE, USA) where a ratio of 1.8–2.0 (OD260/280) is considered acceptable. cDNA was synthesized using HiScipt II Q-RT SuperMix for qPCR (+ qDNA wiper) (Vazyme, Nanjing, China) according to the manufacturer’s instructions. Quantitative real time PCR (qPCR) was conducted using AceQ qPCR SYBR Green Master Mix (Vazyme, Nanjing, China). The sequence of the primers (GADPH, ERb, GPER, CCND1, CDK4, CDK6, Ki67, YAP1, EGFR, TAZ, CYR61, AREG, c-jun, c-fos, Sirt1) are listed in Table S2. All cDNA samples were run on StepOnePlus Real Time PCR system (Applied Biosystems, CA, USA). The relative RNA expressions were normalized against GADPH using 2^-∆∆CT^ method.

### Confocal immunofluorescence staining

The cells were seeded on confocal dishes (1 × 10^5^ cells per dish) (Jet Biofil, Guangzhou, China) After treatment, they were fixed with 4% paraformaldehyde-PBS for 15 min, and then permeabilized with 0.1% Triton X-100 in TBS. After blocking with 10% goat serum in PBS for one hour, the cells were incubated with anti-Ki67 antibody (Abcam, CA, USA) or anti-YAP antibody (Cell Signaling, MA, USA) at 4 °C, overnight. After three washes with PBS, the cells were incubated with Alexa Fluor 488 tagged goat anti-rabbit antibody (Cell Signaling, MA, USA, 1:1000 dilution) for 2 h and counterstained with 4′6′-diamidino-2-phenylindole (DAPI) (Thermo Fisher Scientific, MA, USA) for 5 min at room temperature. Immunofluorescence was detected using Zeiss LSM 710 confocal laser scanning microscope with multiphoton System and analysed with Zeiss LSM software (Carl Zeiss, Germany).

### Western blotting

The treated cells were collected in RIPA buffer (Sigma, MO, USA). Nuclear and cytoplasmic protein extractions were prepared using NE-PER nuclear and cytoplasmic extraction kit (Thermo Scientific, MA, USA) following manufacturer’s instructions. Total protein content was measured by DC protein assay (Bio-Rad, CA, USA). The extracted proteins (20 μg) were then resolved on 10% SDS-PAGE and transferred to PVDF membrane. The membrane was then blocked with 5% skim milk or BSA, followed by overnight incubation with primary antibodies, anti-YAP/TAZ, anti-YAP (D8H1X), anti-Phospho-YAP1 (Ser127), anti-CCND1 and anti-GADPH (Cell Signaling, MA, USA), anti-GPER, anti-ERa and anti-Ki67 (Abcam, CA, USA), and anti-b-actin (Invitrogen, CA, USA) at 1:1000 dilution. After washing, the blots were incubated with goat anti-rabbit IgG (H + L)-HRP conjugate or goat anti-rabbit IgG (H + L)-HRP conjugate (Bio-rad, CA, USA). The protein bands were visualized with enhanced chemiluminescence reagent (Clarity Western ECL Substrate, Bio-Rad, CA, USA). GADPH and b-actin served as loading control.

### Statistical analysis

All analysis was performed by using GraphPad Prism 8.0 (GraphPad Software, CA, USA for Mac). The experimental groups were compared by unpaired Student’s t-test or one way analysis of variance (ANOVA), followed by Tukey's Multiple Comparisons Test. A p-value of < 0.05 was considered statistically significant. All analysis were assessed in three independent experiments. Statistical significance is expressed as ***P < 0.001, **P < 0.01 and *P < 0.05.

## Supplementary Information

Supplementary Information

## References

[1] Bray F (2018). Global cancer statistics 2018: GLOBOCAN estimates of incidence and mortality worldwide for 36 cancers in 185 countries. Ca-a Cancer J. Clin..

[2] Holleczek B (2015). On-going improvement and persistent differences in the survival for patients with colon and rectum cancer across Europe 1999–2007 - Results from the EUROCARE-5 study. Eur. J. Cancer.

[3] Allemani C (2015). Global surveillance of cancer survival 1995–2009: analysis of individual data for 25,676,887 patients from 279 population-based registries in 67 countries (CONCORD-2). Lancet.

[4] Zinedine A (2007). Review on the toxicity, occurrence, metabolism, detoxification, regulations and intake of zearalenone: an oestrogenic mycotoxin. Food Chem. Toxicol..

[5] Lecomte S (2019). Deciphering the molecular mechanisms sustaining the estrogenic activity of the two major dietary compounds zearalenone and apigenin in ER-positive breast cancer cell lines. Nutrients.

[6] Kowalska K (2018). Estrogen receptor α is crucial in zearalenone-induced invasion and migration of prostate cancer cells. Toxins.

[7] Belhassen H (2015). Zearalenone and its metabolites in urine and breast cancer risk: a case-control study in Tunisia. Chemosphere.

[8] Gratz SW (2017). Masked trichothecene and zearalenone mycotoxins withstand digestion and absorption in the upper GI tract but are efficiently hydrolyzed by human gut microbiota in vitro. Mol. Nutr. Food Res..

[9] Zielonka L (2015). Zearalenone in the Intestinal Tissues of Immature Gilts Exposed per os to Mycotoxins. Toxins (Basel).

[10] Abassi H (2016). The mycotoxin zearalenone enhances cell proliferation, colony formation and promotes cell migration in the human colon carcinoma cell line HCT116. Toxicol. Lett..

[11] Lahjouji T (2020). Acute exposure to zearalenone disturbs intestinal homeostasis by modulating the Wnt/beta-catenin signaling pathway. Toxins (Basel).

[12] Yip KY (2017). Combined low-dose zearalenone and aflatoxin B1 on cell growth and cell-cycle progression in breast cancer MCF-7 cells. Toxicol. Lett..

[13] Uzgare AR, Kaplan PJ, Greenberg NM (2003). Differential expression and/or activation of P38MAPK, erk1/2, and jnk during the initiation and progression of prostate cancer. Prostate.

[14] Zhu CF (2011). PI3K/Akt and MAPK/ERK1/2 signaling pathways are involved in IGF-1-induced VEGF-C upregulation in breast cancer. J. Cancer Res. Clin. Oncol..

[15] Luo J, Yu FX (2019). GPCR-Hippo Signaling in Cancer.. Cells.

[16] Nussinov R, Tsai CJ, Jang H (2017). A New View of Pathway-Driven Drug Resistance in Tumor Proliferation. Trends Pharmacol. Sci..

[17] Nussinov R (2016). Oncogenic KRAS signaling and YAP1/beta-catenin: Similar cell cycle control in tumor initiation. Semin. Cell Dev. Biol..

[18] Leroy B (2014). Analysis of TP53 mutation status in human cancer cell lines: a reassessment. Hum. Mutat..

[19] Nakayama M, Oshima M (2019). Mutant p53 in colon cancer. J. Mol. Cell Biol..

[20] Bustos V (2017). GPER mediates differential effects of estrogen on colon cancer cell proliferation and migration under normoxic and hypoxic conditions. Oncotarget.

[21] Eskola M (2020). Worldwide contamination of food-crops with mycotoxins: Validity of the widely cited 'FAO estimate' of 25%. Crit. Rev. Food Sci. Nutr..

[22] Sarkanj B (2018). Ultra-sensitive, stable isotope assisted quantification of multiple urinary mycotoxin exposure biomarkers. Anal. Chim. Acta.

[23] Arber N (1996). Increased expression of cyclin D1 is an early event in multistage colorectal carcinogenesis. Gastroenterology.

[24] Mermelshtein A (2005). Expression of D-type cyclins in colon cancer and in cell lines from colon carcinomas. Br. J. Cancer.

[25] McKay JA (2000). Cyclin D1 protein expression and gene polymorphism in colorectal cancer. Aberdeen Colorectal Initiative. Int J Cancer.

[26] Palaiologos P (2019). The Prognostic Value of G1 Cyclins, p21 and Rb Protein in Patients With Colon Cancer. Anticancer Res.

[27] Luo Z-W (2019). Increased expression of Ki-67 is a poor prognostic marker for colorectal cancer patients: a meta analysis. BMC Cancer.

[28] Vejdovszky K (2016). Non-synergistic cytotoxic effects of Fusarium and Alternaria toxin combinations in Caco-2 cells. Toxicol. Lett..

[29] Campbell-Thompson M, Lynch IJ, Bhardwaj B (2001). Expression of estrogen receptor (ER) subtypes and ERbeta isoforms in colon cancer. Cancer Res..

[30] Arai N (2000). Estrogen receptor beta mRNA in colon cancer cells: growth effects of estrogen and genistein. Biochem. Biophys. Res. Commun..

[31] Gilligan LC (2017). Estrogen Activation by Steroid Sulfatase Increases Colorectal Cancer Proliferation via GPER. J. Clin. Endocrinol. Metab..

[32] Liu Q (2017). Epigenetic down regulation of G protein-coupled estrogen receptor (GPER) functions as a tumor suppressor in colorectal cancer. Mol. Cancer.

[33] Wei Y (2019). Estrogen Receptor Beta (ERbeta) Mediated-CyclinD1 Degradation via Autophagy Plays an Anti-Proliferation Role in Colon Cells. Int. J. Biol. Sci..

[34] Xie M (2019). Low Doses of Nonylphenol Promote Growth of Colon Cancer Cells through Activation of ERK1/2 via G Protein-Coupled Receptor 30. Cancer Res..

[35] Grossi V (2014). p38alpha MAPK pathway: a key factor in colorectal cancer therapy and chemoresistance. World J. Gastroenterol..

[36] de Valdivia EG (2017). G protein-coupled estrogen receptor 1 (GPER1)/GPR30 increases ERK1/2 activity through PDZ motif-dependent and -independent mechanisms. J. Biol. Chem..

[37] Harvey KF, Zhang X, Thomas DM (2013). The Hippo pathway and human cancer. Nat. Rev. Cancer.

[38] Wang Y (2013). Clinical and prognostic significance of Yes-associated protein in colorectal cancer. Tumour. Biol..

[39] Shreberk-Shaked M, Oren M (2019). New insights into YAP/TAZ nucleo-cytoplasmic shuttling: new cancer therapeutic opportunities?. Mol. Oncol..

[40] Wang Z (2017). Endothelin Promotes Colorectal Tumorigenesis by Activating YAP/TAZ. Can. Res..

[41] Zhou X (2015). Estrogen regulates Hippo signaling via GPER in breast cancer. J. Clin. Invest.

[42] Reddy B, Irvine KD (2013). Regulation of Hippo signaling by EGFR-MAPK signaling through Ajuba family proteins. Dev. Cell.

[43] You B (2015). Inhibition of ERK1/2 down-regulates the Hippo/YAP signaling pathway in human NSCLC cells. Oncotarget.

[44] Zhang RQ (2018). Zearalenone exposure elevated the expression of tumorigenesis genes in mouse ovarian granulosa cells. Toxicol Appl Pharmacol.

[45] Yu FX (2012). Regulation of the Hippo-YAP pathway by G-protein-coupled receptor signaling. Cell.

[46] Yu FX, Zhao B, Guan KL (2015). Hippo Pathway in Organ Size Control, Tissue Homeostasis, and Cancer. Cell.

[47] Nowakowska M (2014). Diverse effect of WWOX overexpression in HT29 and SW480 colon cancer cell lines. Tumour. Biol..

[48] Yuan F (2019). A New Regulatory Mechanism Between P53 And YAP Crosstalk By SIRT1 Mediated Deacetylation To Regulate Cell Cycle And Apoptosis In A549 Cell Lines. Cancer Manag. Res..

[49] Hunter MR, Glass M (2015). Increasing the flexibility of the LANCE cAMP detection kit. J. Pharmacol. Toxicol. Methods.

