# Supplementary material for: Low dose of zearalenone elevated colon cancer cell growth through G protein-coupled estrogenic receptor
Source: Sci Rep. 2021 Apr 1;11:7403. doi: 10.1038/s41598-021-86788-w (PMC8016995; doi:10.1038/s41598-021-86788-w)

Supplementary document

Low dose of zearalenone elevated colon cancer cell growth through G protein-coupled estrogenic receptor

Emily Kwun Kwan Lo[1], Jetty Chung-Yung Lee[1], Paul C Turner[2*] and Hani El-Nezami [1,3*]

[1]School of Biological Sciences, the University of Hong Kong, Hong Kong SAR, China;
[2]Maryland Institute for Applied Environmental Health, School of Public Health, University of Maryland, College Park, Maryland, USA
[3]Institute of Public Health and Clinical Nutrition, University of Eastern Finland, Kuopio, Finland
* Correspondence: elnezami@hku.hk, pturner3@umd.edu

Figure S1. Cytotoxic effect of ZEA on human CRC cells. (A) Cultured colon cancer line (SW480, HCT116, HT29) and normal colon cells (CCD841) were treated with 0-100 μM ZEA for 24, 48 and 72 hours. Cell viability was determined by MTT assay, n=5. (B) HCT116 were treated with 0-3 μM ZEA for 24, 48 and 72 h. CRCs were then trypsinized and fixed with trypan blue solution. Cell counting was carried out using LUNA Automated Cell Counter. Results shown are mean ± SEM, n=3. *p<0.05 and **p<0.01 compared to control.

A

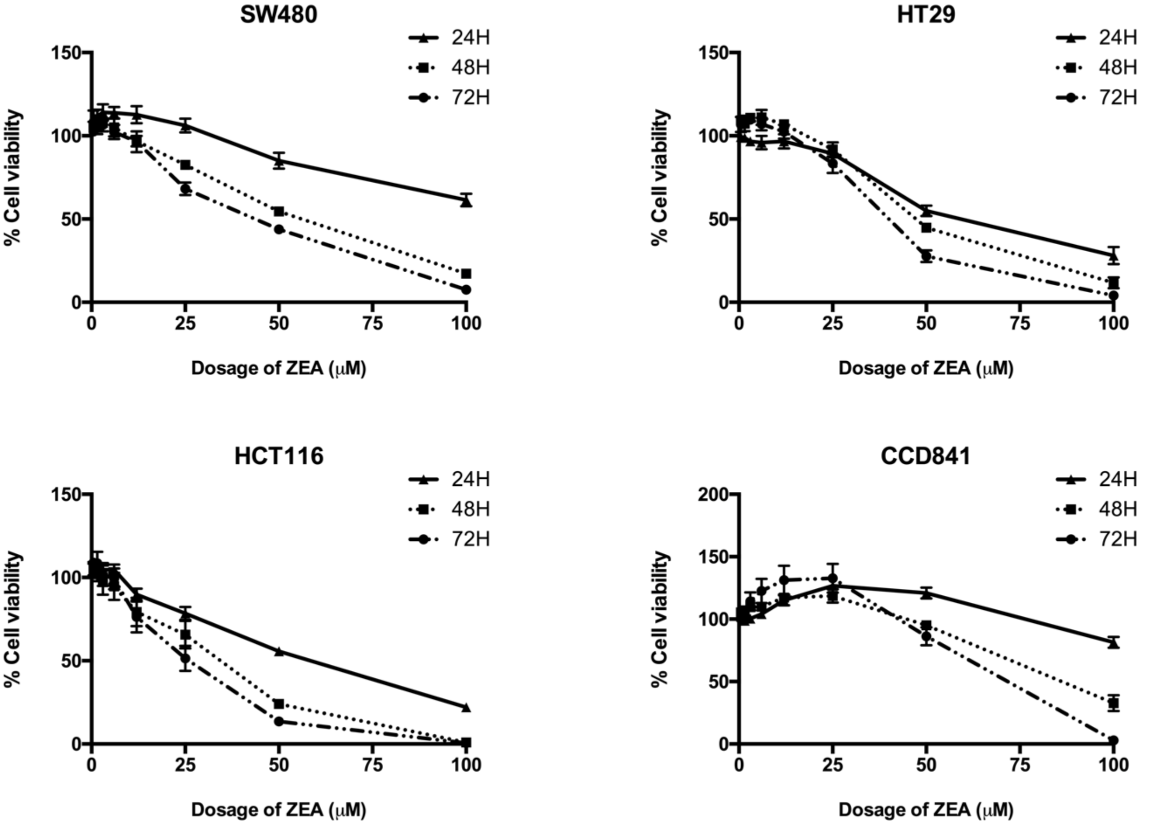

B

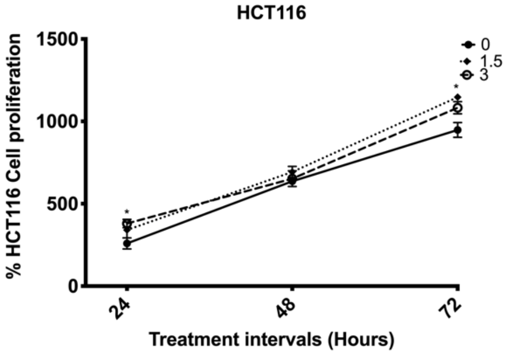

Figure S2. Cell cycle analysis of SW480 and HT29 with or without 3 μM ZEA treatment for 24 hours. The percentage of S phase, G0/G1 and G2/M are shown in the figures. Results shown are mean ± SEM, with n=3. *p<0.05, **p<0.01 and ***p<0.001.

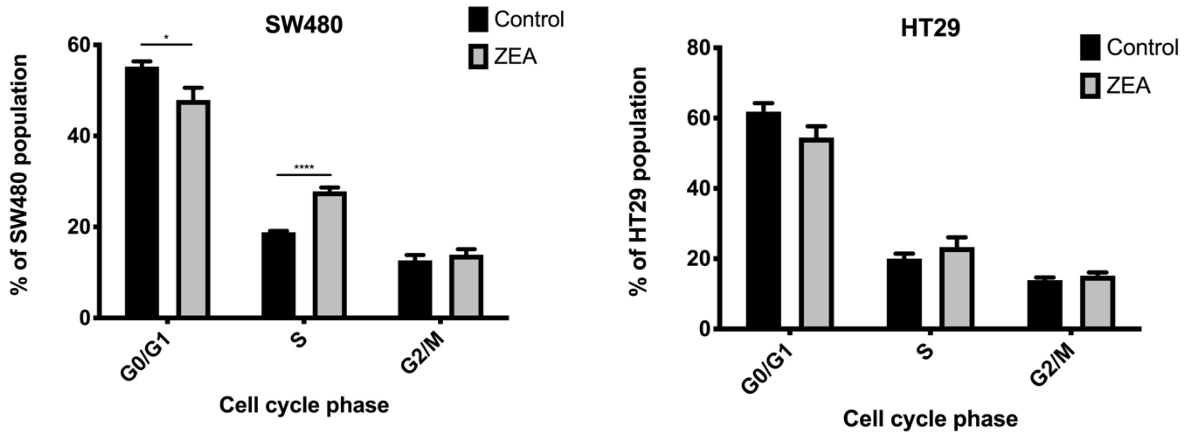

Figure S3. Effect of ZEA on GPER expression in SW480 and HT29 cell line. (A) Basal protein expression of GPER in SW480 and HT29 cells were determined by western blotting. (B) Basal protein expression of ERα in SW480 and HT29 cells were determined by western blotting. Protein from MCR-7 cells which known to present ERα represent the positive control. (C) Western blot analysis of GPER protein expression in SW480 and HT29 cells with and without 3 μM ZEA was investigated. Results shown are mean ± SEM, with n=3. *p<0.05 and ****p<0.0001.

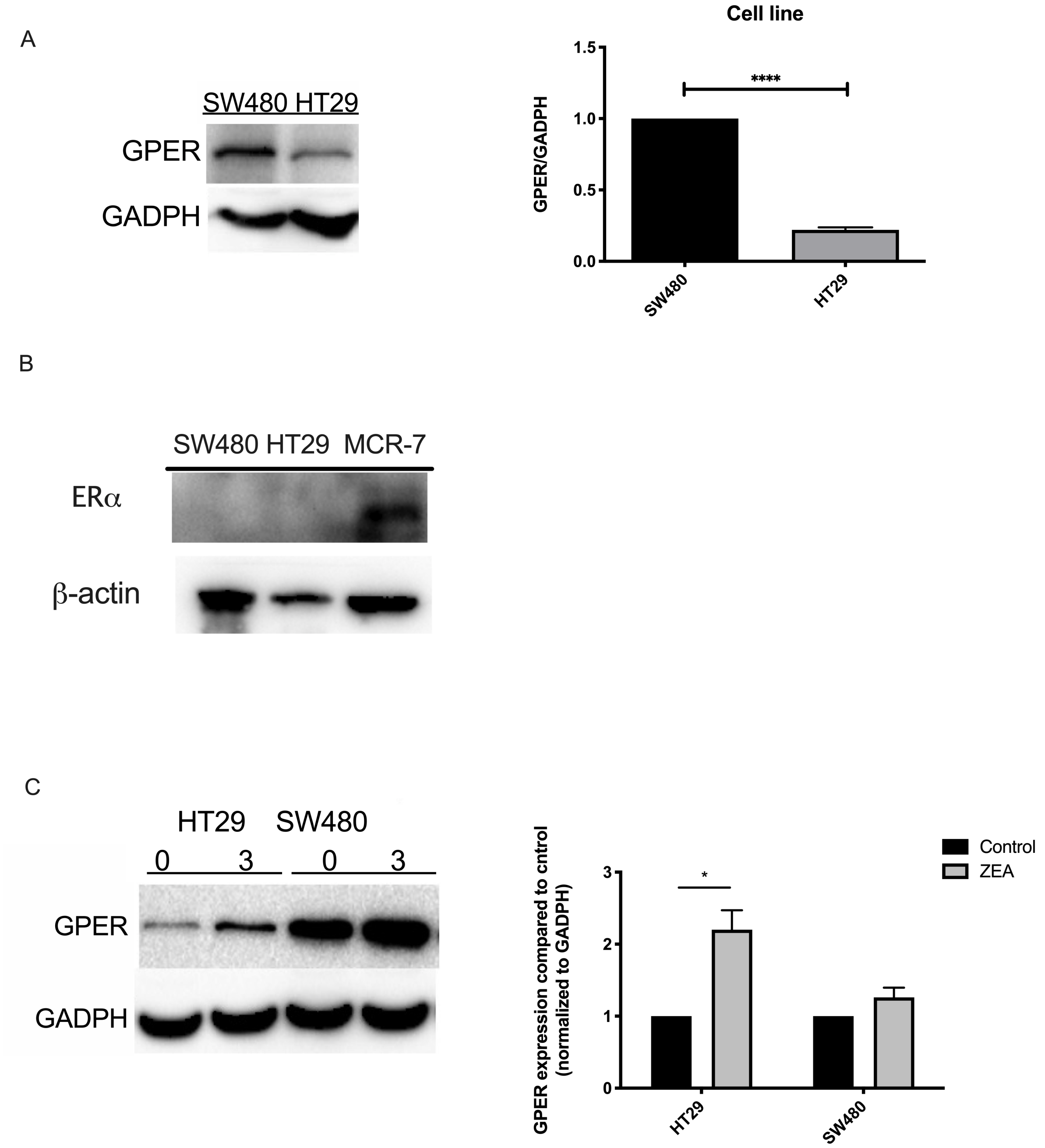

Figure S4. ZEA promoted YAP gene expression. RT-qPCR analysis of YAP1, TAZ and downstream marker CYR61 expression in SW480 and HT29 with and without 3 μM ZEA treatment for 24 h.

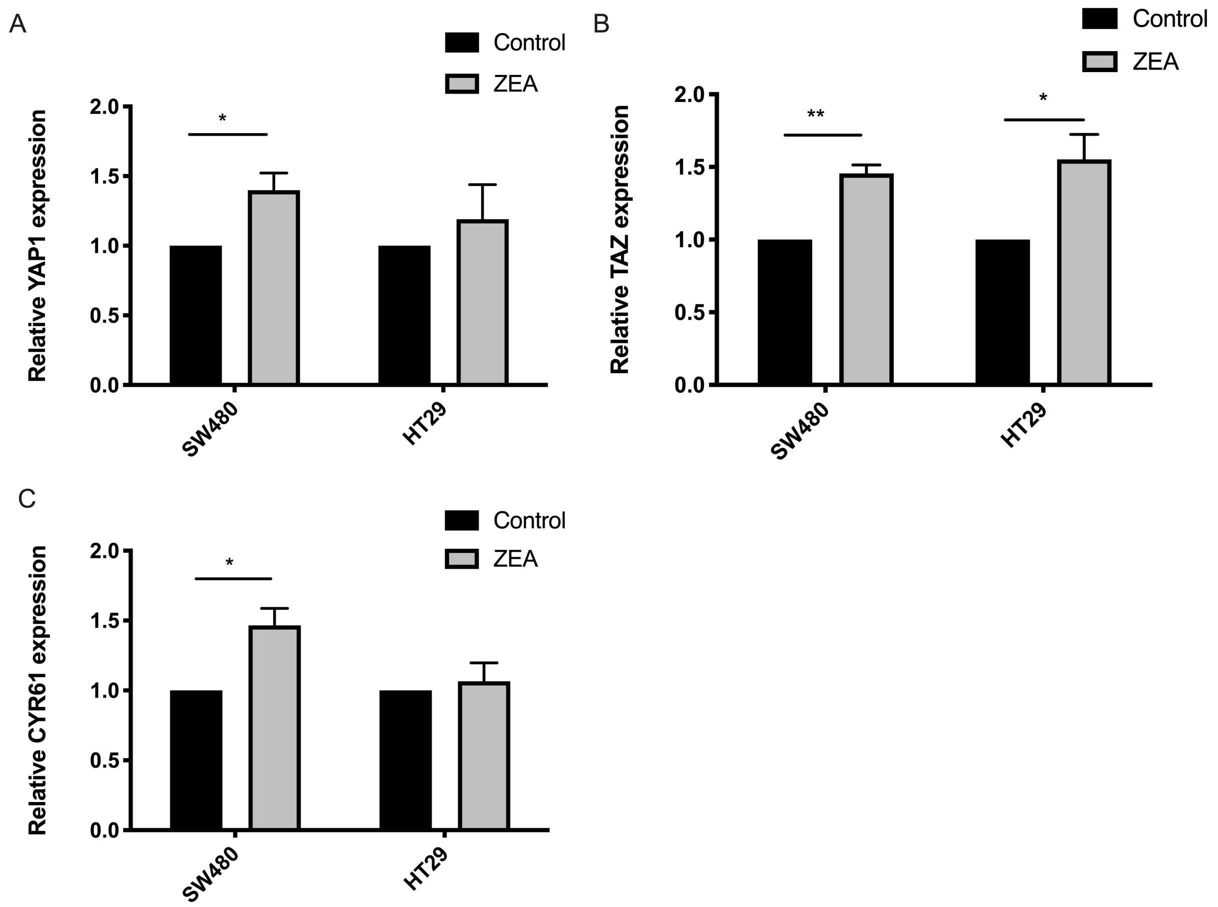

Figure S5. Cell cycle analysis of SW480 and HT29 co-incubated 3 μM ZEA with or without G15 for 24 h. Representative figure of FITC-BrdU/7-AAD cell cycle analysis.

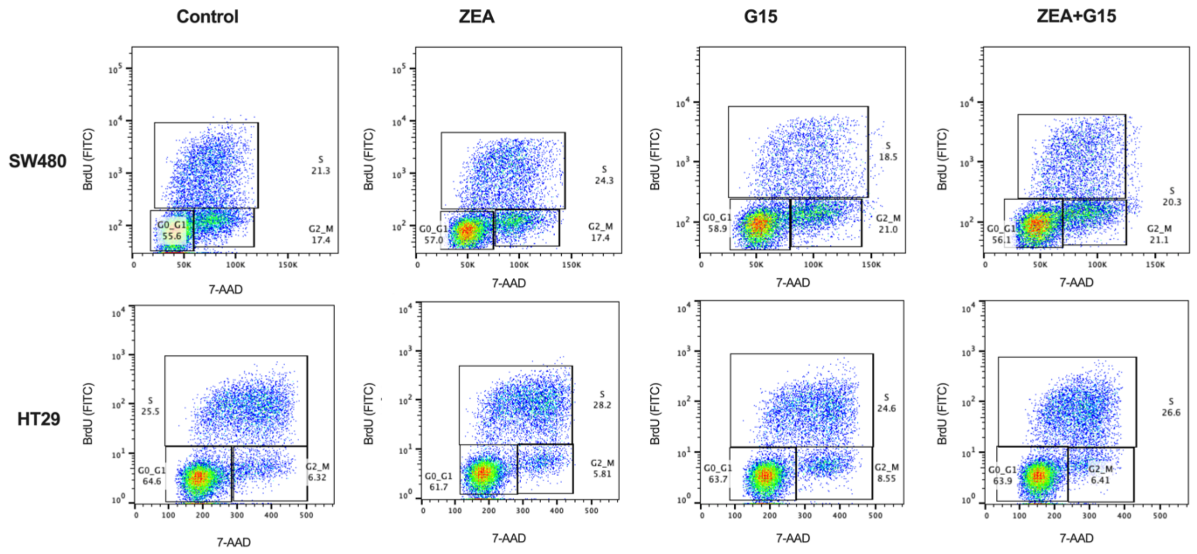

Table S1. Characteristic of the Cell lines used in the experimentation. [1]

Cell line	APC mutation	β-Catenin mutation	K-Ras	p53
**HT-29**	1 bp insertion (codon 1555)	Wild type	Wild type	R273H
**SW480**	CAG-TAG (stop) (codon 1338)	Wild type	G12V	R273H;P309S

Table S2. Nucleotide sequences of the primers used for the qPCR analysis

Primer set	GenBank	Forward primer sequence (5'->3')	Reverse primer sequence (3'->5')
GAPDH	NM_001256799.3	CCAGCAAGAGCACAAGAGGAAGAG	CAAGGGGTCTACATGGCAACTGTG
ERβ	NM_001291723.1	TTCCAGGCAGTAATGGGCG	CGCAGGTGTGTGACAAAGTC
GPER	NM_001505.3	TCAACCTCGCCGCCTTCTCCAA	CGAGGAGCCAGAAGCCACATC
CCND1	NM_053056.3	TGAGGGACGCTTTGTCTGTC	GCCTTTGGCCTCTCGATACA
CDK4	NM_000075.4	GTGTATGGGGCCGTAGGAAC	CCATAGGCACCGACACCAAT
CDK6	NM_001259.7	GCAGGGAAAGAAAAGTGCAATGA	TCCTCGAAGCGAAGTCCTCA
Ki67	NM_002417.5	CGTCCCAGTGGAAGAGTTGT	CGACCCCGCTCCTTTTGATA
YAP1	NM_001195045.2	TAGCCCTGCGTAGCCAGTTA	TCATGCTTAGTCCACTGTCTGT
EGFR	NM_005228.5	GACAGGCCACCTCGTCG	CCGGCTCTCCCGATCAATAC
TAZ	NM_000116.5	GGACCAAGTACATGAACCACC	GACTGGTGATTGGACACGGT
CYR61	NM_001554.4	CGCCTTGTGAAAGAAACCCG	GGTTCGGGGGATTTCTTGGT
AREG	NM_001657.3	TGTCGCTCTTGATACTCGGC	AGGCATTTCACTCACAGGGG
c-jun	NM_002228.3	GAGCTGGAGCGCCTGATAAT	CCCTCCTGCTCATCTGTCAC
c-fos	NM_005252.3	TACTACCACTCACCCGCAGA	TGAAGTTGGCACTGGAGACG
Sirt1	NM_012238.5	TGGCAAAGGAGCAGATTAGTAGG	CTGCCACAAGAACTAGAGGATAAGA

References:

1. Ahmed, D., et al., *Epigenetic and genetic features of 24 colon cancer cell lines.* Oncogenesis, 2013. **2**: p. e71.

Supplementary information: Full blot images of western blot data

Low dose of zearalenone elevated colon cancer cell growth by regulating HIPPO pathway and activating ERK1/2 through G Protein-Coupled Receptor 30

Emily Kwun Kwan Lo[1], Jetty Chung-Yung Lee[1], Paul C Turner[2]* and Hani El-Nezami [1,3]*

[1]School of Biological Sciences, the University of Hong Kong, Hong Kong SAR, China;
[2]Maryland Institute for Applied Environmental Health, School of Public Health, University of Maryland, College Park, Maryland, USA
[3]Institute of Public Health and Clinical Nutrition, University of Eastern Finland, Kuopio, Finland
* Correspondence: elnezami@hku.hk, pturner3@umd.edu

Supplementary Figure S4. Full-length gels from Figures

Some membranes were used for several samples and were cut, so only the portion containing the described samples and bands would be visualized

Figure 2B

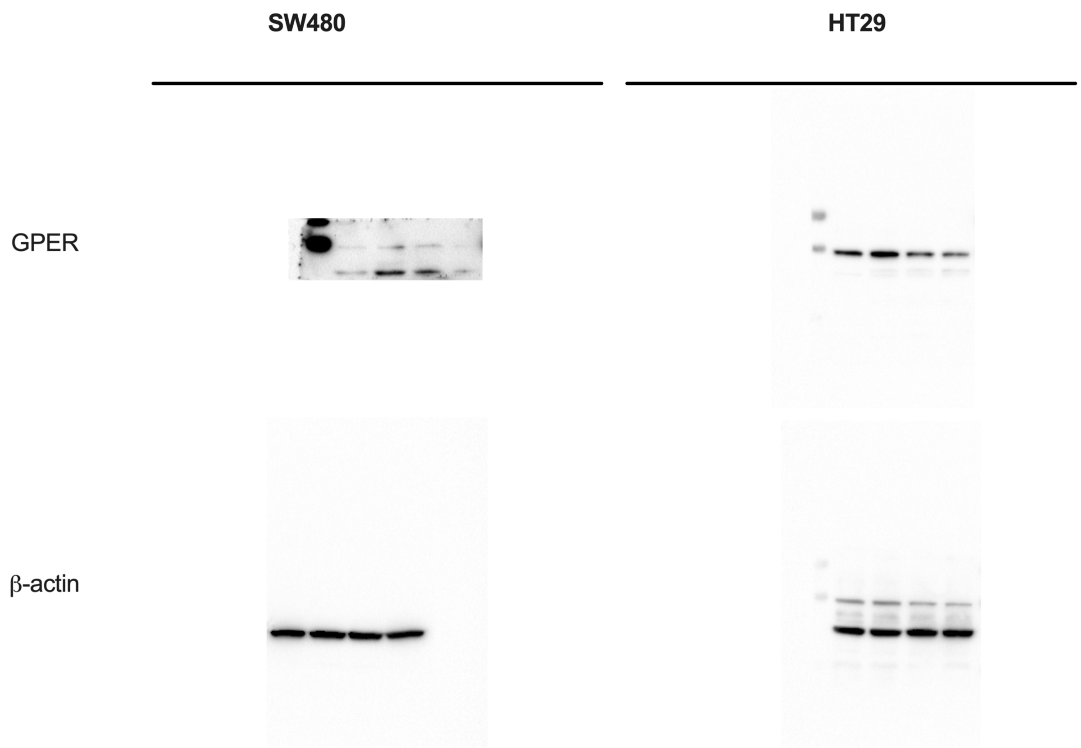

Figure 4D

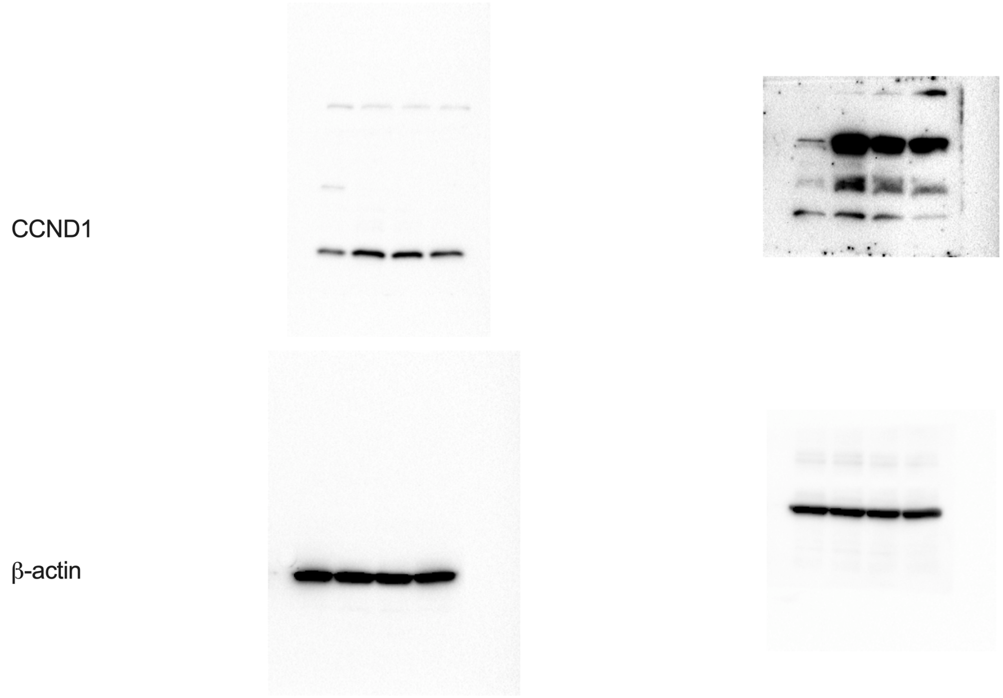

Figure 5A

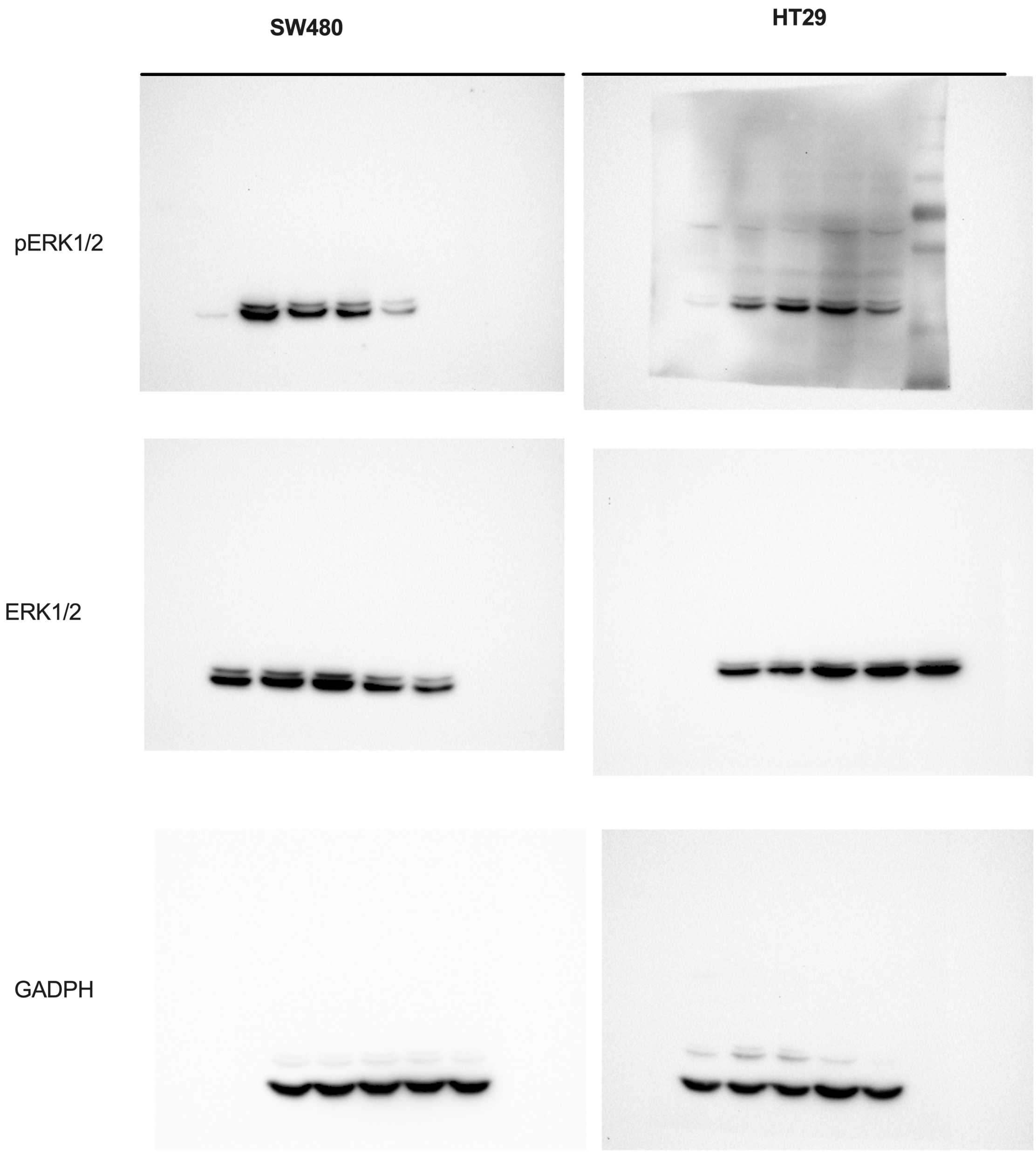

Figure 5B

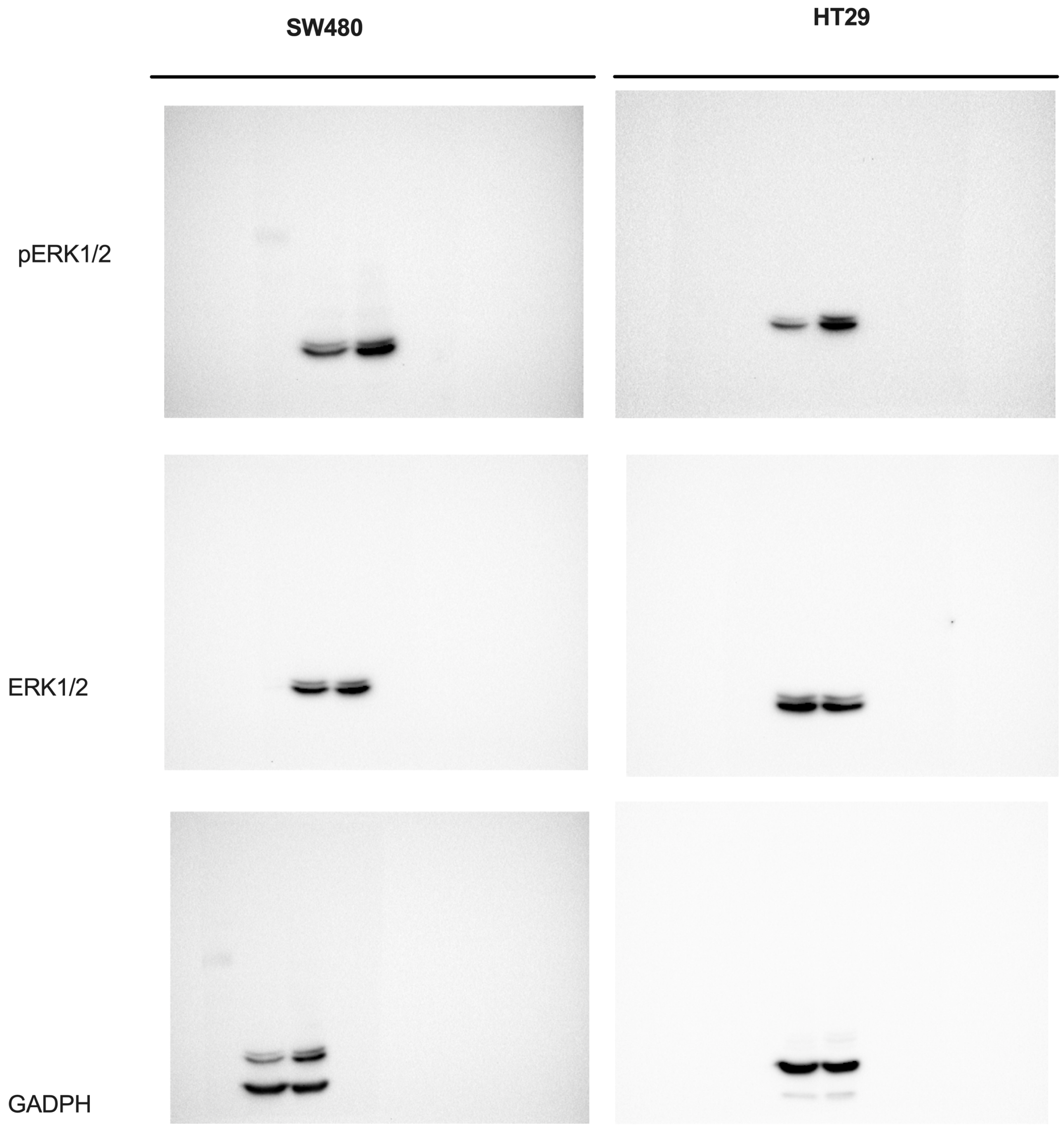

Figure 6A

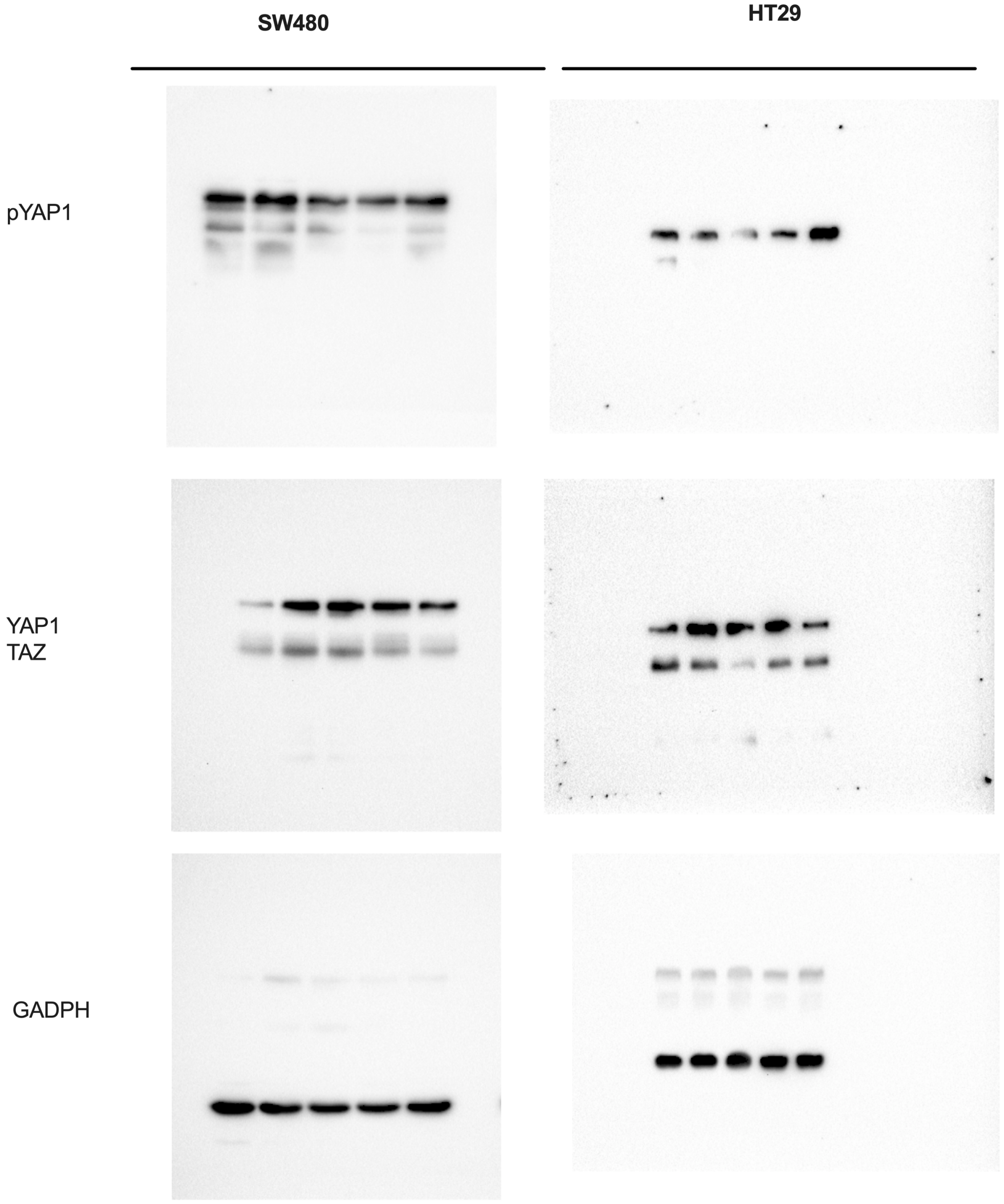

Figure

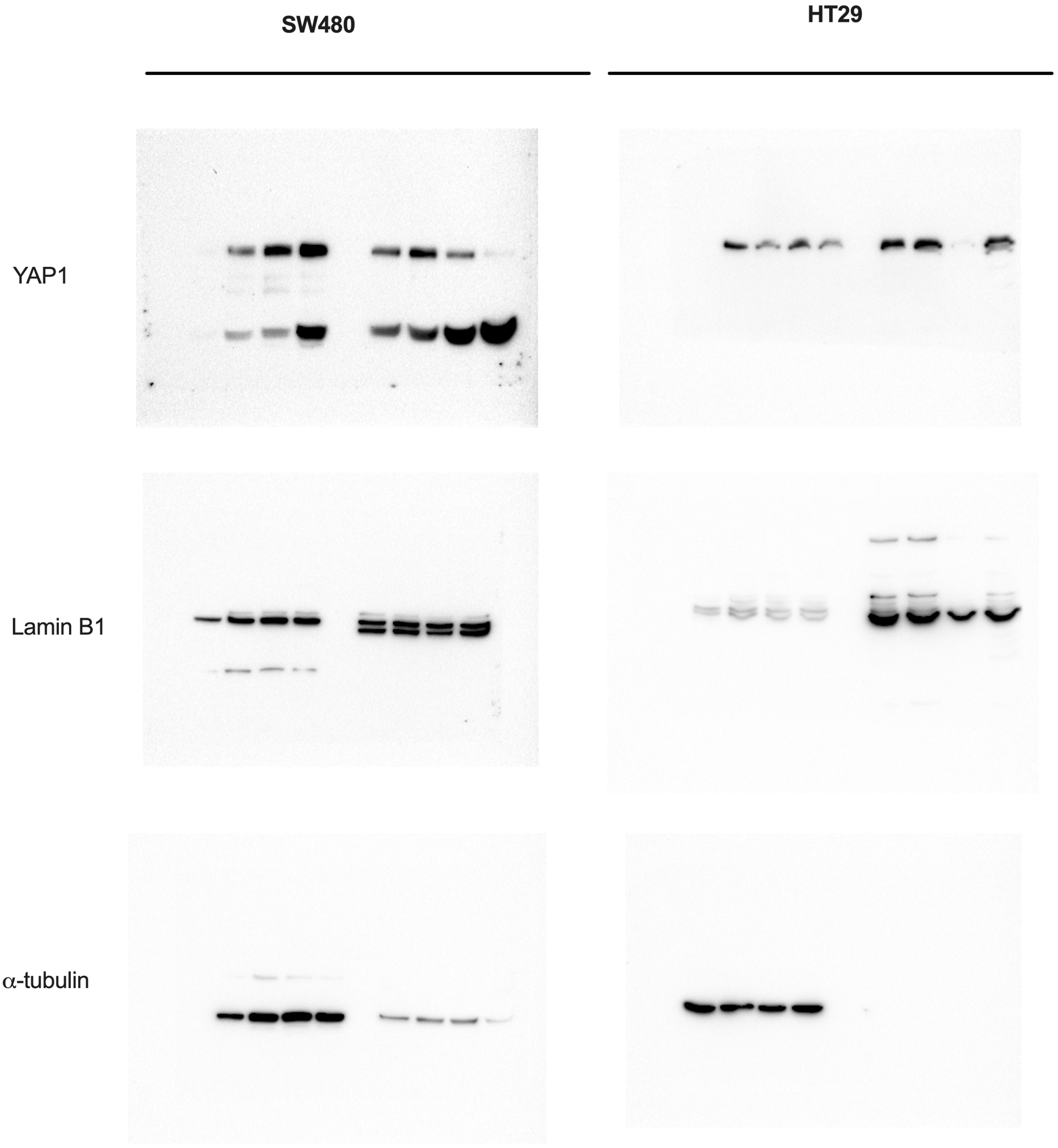

Figure 6B

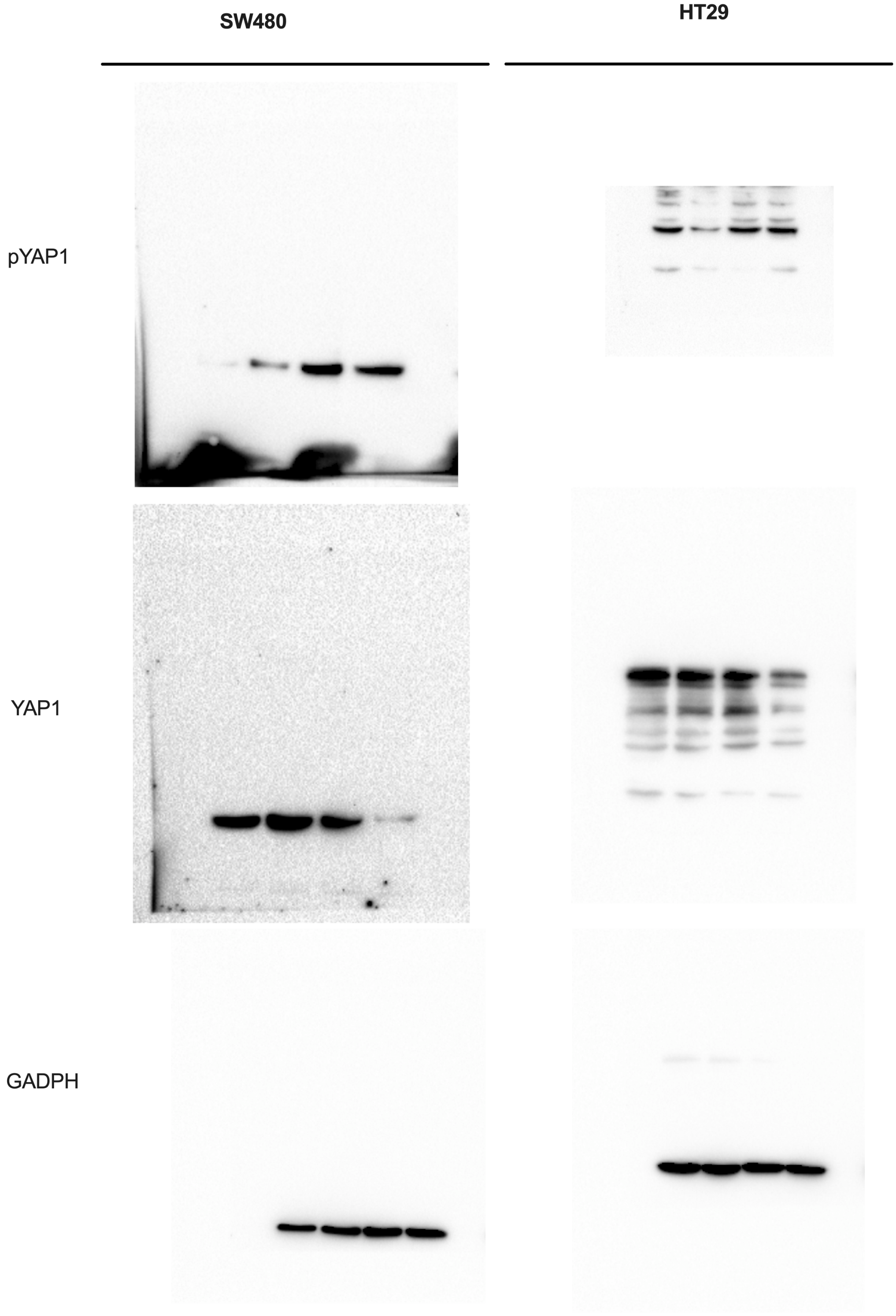

Figure S3A

GPER

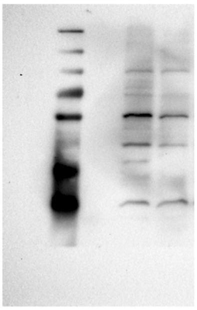

GADPH

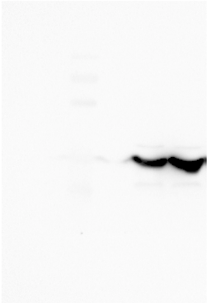

Figure S3B

ERα

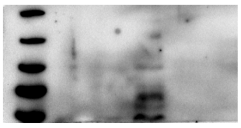

β-actin

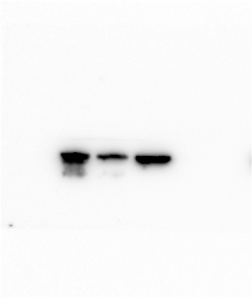

Figure S3c

GPER

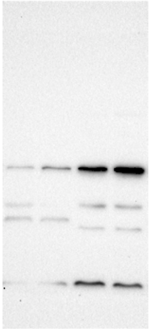

GADPH

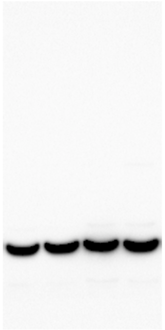

Supplement: Supplementary file 1 — Supplementary Information [file 41598_2021_86788_MOESM1_ESM.pdf]
